# Mass Spectrometry-Based Characterization of New Spirolides from *Alexandrium ostenfeldii* (Dinophyceae)

**DOI:** 10.3390/md18100505

**Published:** 2020-10-02

**Authors:** Joyce A. Nieva, Jan Tebben, Urban Tillmann, Sylke Wohlrab, Bernd Krock

**Affiliations:** 1 Alfred Wegener Institut-Helmholtz Zentrum für Polar- und Meeresforschung, Ökologische Chemie, Am Handelshafen 12, 27570 Bremerhaven, Germany; joyce.nieva@awi.de (J.A.N.); Jan.Tebben@awi.de (J.T.); urban.tillmann@awi.de (U.T.); sylke.wohlrab@awi.de (S.W.); 2 Helmholtz Institut für Funktionelle Marine Biodiversität an der Universität Oldenburg (HIFMB), Ammerländer Heerstraße 231, 26129 Oldenburg, Germany

**Keywords:** CID spectrum, fragmentation pattern, cyclic imine, elemental composition, triketal ring system, proposed structure

## Abstract

Spirolides belong to a group of marine phycotoxins produced by the marine planktonic dinophyte *Alexandrium ostenfeldii*. Composed of an imine moiety and a spiroketal ring system within a macrocylcle, spirolides are highly diverse with toxin types that vary among different strains. This study aims to characterize the spirolides from clonal *A. ostenfeldii* strains collected from The Netherlands, Greenland and Norway by mass spectral techniques. The structural characterization of unknown spirolides as inferred from high-resolution mass spectrometry (HR-MS) and collision induced dissociation (CID) spectra revealed the presence of nine novel spirolides that have the pseudo-molecular ions *m/z* 670 (**1**), *m/z* 666 (**2**), *m/z* 696 (**3**), *m/z* 678 (**4**), *m/z* 694 (**5**), *m/z* 708 (**6**), *m/z* 720 (**7**), *m/z* 722 (**8**) and *m/z* 738 (**9**). Of the nine new spirolides proposed in this study, compound **1** was suggested to have a truncated side chain in lieu of the commonly observed butenolide ring in spirolides. Moreover, there is indication that compound **5** might belong to new spirolide subclasses with a trispiroketal ring configuration having a 6:5:6 trispiroketal ring system. On the other hand, the other compounds were proposed as C- and G-type SPX, respectively. Compound **7** is proposed as the first G-type SPX with a 10-hydroxylation as usually observed in C-type SPX. This mass spectrometry-based study thus demonstrates that structural variability of spirolides is larger than previously known and does not only include the presence or absence of certain functional groups but also involves the triketal ring system.

## 1. Introduction

In the past four decades, there has been widespread occurrence of harmful algal blooms (HABs) in many coastal areas. HABs can have noxious or nuisance effects on the environment, causing, e.g., oxygen depletion or foam formation, and also may cause detrimental effects to the public health and economy [1]. HAB species that are considered toxic may cause major fish mortality or their toxin may accumulate in seafood [2]. One group of these toxins are the spiroimine shellfish toxins [3]. Spirolides (SPX) were first identified during diarrhetic shellfish poisoning (DSP) toxin profiling of shellfish (*Mytilus edulis*) from aquaculture sites in the Atlantic Coast of Nova Scotia, Canada. Despite low concentrations of DSP toxins, a rapid mortality in mice was observed. The unusual behavior in the mouse bioassay led to the conclusion that fast-acting toxins with neurotoxic potential might have been present, which were later identified as SPX [4]. The causative microorganism producing the toxin was subsequently referred to as *Alexandrium ostenfeldii* [5], a planktonic dinophyte first described in the early 20th century from the north-east coast of Iceland [6].

Since the discovery of SPX in 1995 [3], more information has been reported on the global distribution and types of spirolides. SPX, which belong to the macrocyclic marine phycotoxin group called cyclic imines, are represented by a bicyclic ring system composed of a cyclic imine moiety and a 5:5:6 trispiroketal ring system (Table 1). The first characterized SPX, which were gathered from Canadian microalgal and contaminated shellfish samples, included spirolides A-D and the 13-desmethyl analogue of SPX C (=SPX 1) [3,7] and D [8]. The 5:5:6 trispiroketal ring system spirolides were also observed from microalgal samples from the Adriatic coast [9,10] and as a structural feature of recently elucidated new spirolides from *A. ostenfeldii* strains from The Netherlands [11]. In addition, new spirolide subclasses, G-type [12] and SPX H/I [13] were discovered. In contrast to C-type SPX, G-type spirolides have a 5:6:6 trispiroketal ring configuration and H-type SPX contain a 5:6 dispiroketal ring system (Table 1).

The occurrence of SPX in *A. ostenfeldii* strains has also been observed across European waters [12,14,15,16,17,18,19,20,21], in the Gulf of Maine [22] and the South American Atlantic and Pacific coasts [23,24,25] demonstrating that there is high variability of SPX among microalgal strains. To date, 15 SPX of microalgal origin have been structurally elucidated (Table 1) and many others identified based on their characteristic collision induced dissociation (CID) spectra from various regions across the world. While some strains primarily produce SPX 1 [7,9,20,26], other strains have SPX A [22], SPX C [17], 20-methyl SPX G [12,26] or 13,19-didesmethyl SPX C [15] as their major component. Next to their major SPX, the vast majority of strains also contain minor constituents, which hardly have been characterized. This indicates that there is a high chemical variability of SPX and also an intraspecific variability of toxin profiles among *A. ostenfeldii* strains.

Despite increasing knowledge on the distribution and structural diversity of SPX, less is known about their bioactivity and a potential ecological function of SPX to the producing cells is completely obscure. To gain a better understanding on the bioactivity of SPX, mouse toxicity and inhibition assays were performed using animal models [27,28] and in vitro assays [29,30], respectively. Notably, SPX were found to be less toxic in oral administration as compared to intraperitoneal injection treatments [28]. For this reason, it is still under debate to which extent SPX might pose a risk to human health, especially as so far no adverse effect on humans have been reported to be caused by SPX [31]. On the other hand, the mode of action of SPX using inhibition assays described SPX as anticholinergic to acetylcholine receptors [29,30]. Succeeding bioactivity studies on SPX also focused on the structure–activity relationship to reveal links between the different types of SPX and their underlying activity. These studies have shown that the potent neurotoxic activity of SPX was due to distinct chemical features—the cyclic imine moiety and the trispiroketal ring system [15,27,28]. Based on the different toxic activity between the cyclic imines and their hydrolyzed forms, in which the imine group is hydrolyzed to a primary amine, the cyclic imine moiety was postulated as the fundamental pharmacophore. The hydrolyzed form of SPX A and B are SPX E and F, which are formed by shellfish metabolism, while the former are of microalgal origin [4]. Aside from the cyclic imine moiety, the spiroketal ring configuration was also suggested to contribute to spirolide toxicity. Despite having an intact cyclic imine moiety, SPX H and I were inactive in mouse bioassays, implying that the cyclic imine may not be the only pharmacophore of SPX [13]. While two distinct chemical features of SPX causing toxicity are thus identified, there is still a need to search for other relevant pharmacophores of spirolides.

In natural product chemistry, nuclear magnetic resonance (NMR) is the only technique for unambiguous structural elucidation of novel compounds. This method is a non-destructive technique and gives absolute structural configuration; yet high amounts of pure compound are required. Tandem mass spectroscopy coupled to liquid chromatography (LC-MS/MS), as a complementary technique, can provide partial structural information and requires only several orders of magnitude lower amounts of samples due to its lower limit of detection relative to NMR. As the requirement of relatively high amounts of pure substances poses a severe limitation in many cases, much of our understanding on the structural variability of phycotoxins is based on mass spectral data [12,21,32,33]. LC-tandem high-resolution mass spectrometry (LC-HRMS/MS) has successfully complemented low resolution MS in the screening for unknown phycotoxins as well as in the characterization of structural information when the toxin concentration is too low for NMR analysis [34]. The structural information of tandem mass spectrometry depends on the fragmentation of the compounds and, for this reason, only structural proposals including several options can be deduced within a certain fragment. Examples in the case of SPX were the description of SPX variability among *A. ostenfeldii* strains as well as structural characterization acquired through mass spectrometry being conducted from strains isolated from Canada [8], The Netherlands [21] and Argentina [25]. The aim of this work was to add knowledge on the structural SPX variability of *A. ostenfeldii* strains isolated from recent surveys in The Netherlands, Greenland and Norway by mass spectral characterization.

## 2. Results and Discussion

### 2.1. Interpretation of Collision-Induced Dissociation (CID) Spectra

The structural characterization of unknown compounds was conducted by comparing the fragmentation pattern described from the collision-induced dissociation (CID) spectra of the unknown analyte with that of a related compound with known structures.

**Figure 1:**
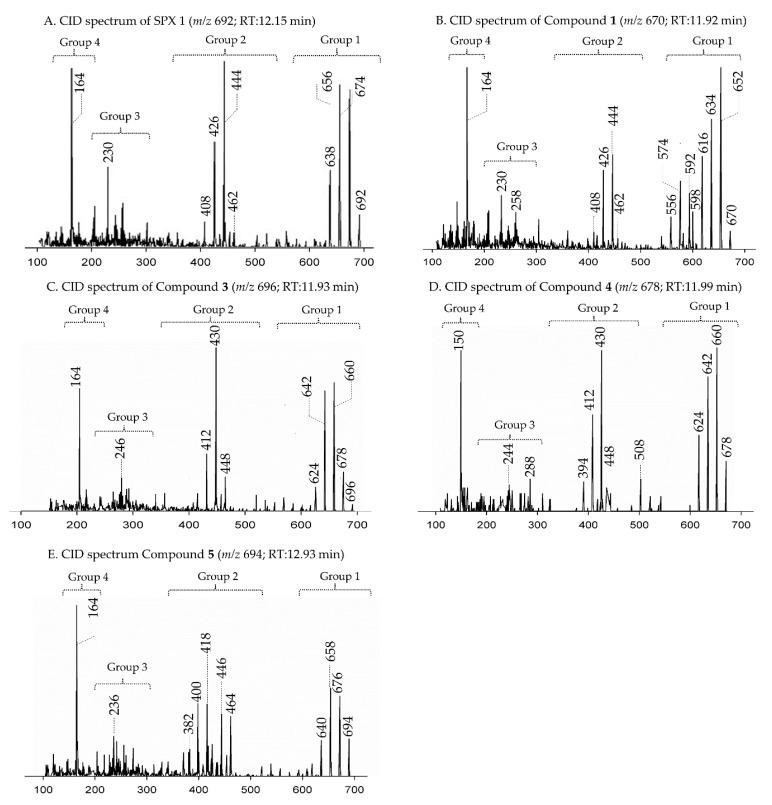
Collision-induced (CID) spectra of novel spirolides (SPX) detected in *A. ostenfeldii* strains related to C-type spirolides: (**A**) SPX 1; (**B**) Compound **1**; (**C**) Compound **3**; (**D**) Compound **4**; and (**E**) Compound **5**.

**Figure 2:**
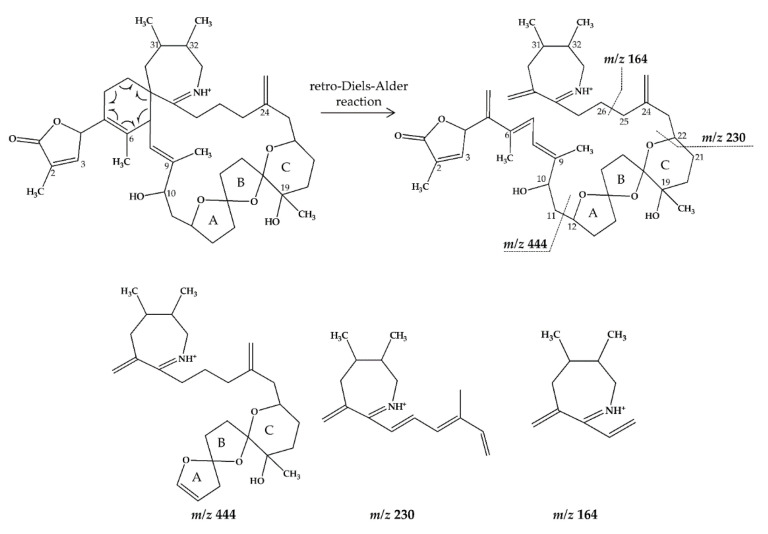
Chemical structure and fragmentation pattern of SPX 1 (modified from [36]). Dashed lines indicate the cleavage sites resulting in the corresponding fragments.

**Figure 3:**
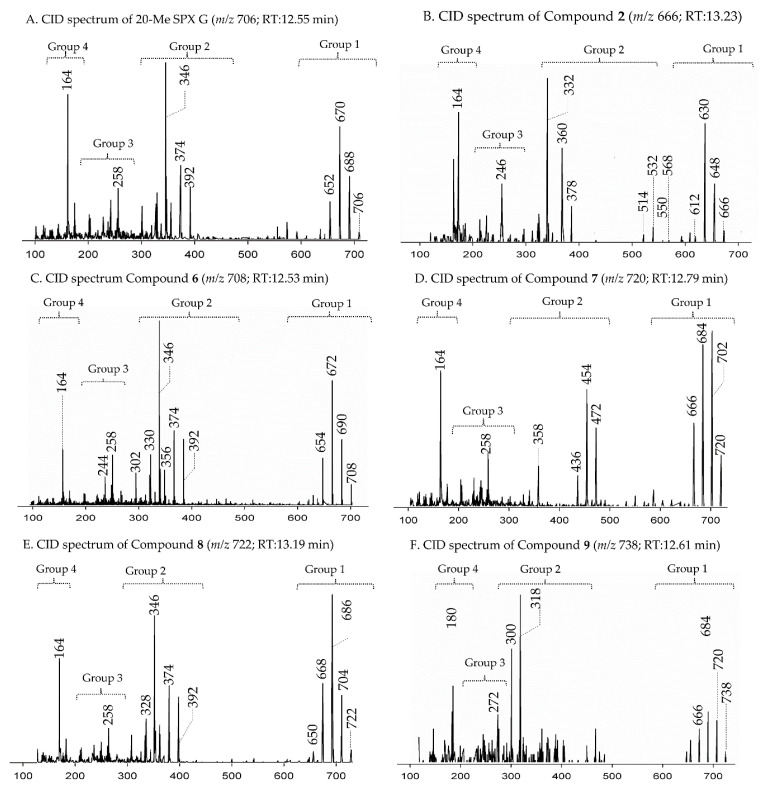
Collision-induced (CID) spectra of novel SPX detected in *A. ostenfeldii* strains related to G-type spirolides: (**A**) 20-Me SPX G; (**B**) Compound **2**; (**C**) Compound **6**; (**D**) Compound **7**; (**E**) Compound **8**; and (**F**) Compound **9**.

In CID spectra, the pseudo-molecular ion ([M + H]^+^) of the compound in the CID spectrum is typically described as the peak with the highest mass-to-charge (*m/z*) ratio. In the case of 13-desmethyl spirolide C (SPX 1), this is the pseudo-molecular ion *m/z*, 692 (Figure 1A), which represented the unfragmented form of the compound (Figure 2). As the pseudo-molecular ion is fragmented in the collision cell, stable products including charged and neutral species are formed after the cleavage of the weakest bonds [35]. For compounds that possess a cyclohexenyl moiety, the fragmentation is preceded by the opening of the macrocyclic system through retro-Diels-Alder (RDA) reaction, which converts the macrocyclic molecule into its linear form [36]. As shown in SPX 1, the fragment cluster produced by a RDA reaction and subsequent water losses, which are typically observed in polyether compounds such as SPX, is named group 1 cluster [36]. The fragment cluster caused by the fragmentation at C11-C12 (and subsequent water losses) is named group 2 cluster (e.g., *m/z* 444 in SPX 1, Figure 2). Corresponding to the formation of the charged fragment *m/z* 444, a neutral loss (NL) of many C-type SPX that have a 5:5:6 (ring A, B, C, respectively) trispiroketal ring system occurs. A rather rare fragmentation can often be observed for SPX involving the C11-C12 cleavage. In SPX 1, a fragment is formed with *m/z* 462 at a lower intensity than *m/z* 444 (difference of H2O). This fragment is most likely formed by the protonation of the ether oxygen stabilized by the neighboring hydroxyl group and subsequent charge mediated fragmentation of the C11/12 bond (OH transfer from C10 to C12 and C11/12 cleavage [3]). Equivalent fragments were also observed for other SPX with a C10 hydroxyl group (*m/z* 464, [11]). SPX G, which is isobaric with SPX 1 (same molecular mass), in contrast displays a different fragmentation pattern. The most abundant fragments of this compound (*m/z* 378, 334 and 332) result from the C16–C17 cleavage of its second ketal (6-membered) ring B [12]. Interestingly, no C11/12 cleavage is observed in either SPX G or 20 Me SPX G, which are the only known SPX with no hydroxyl group at C10. This suggests that the C10 hydroxyl group is required for the C11/12 cleavage as well as the *m/z* 462 formation. The smallest high abundant fragment of SPX usually is *m/z* 164 (group 4), consisting of the cyclo imine ring (Figure 2), but can be shifted to *m/z* 150 [7] or 180 [8,10] (Figure 1 and Figure 3) depending on the degree of methylation or hydroxylation or both.

**Figure 4:**
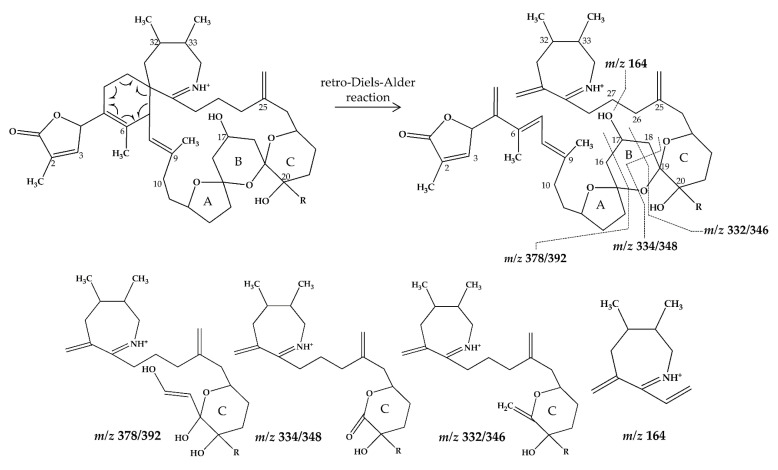
Chemical structure and fragmentation pattern of spirolide G (R=H) and 20-methyl spirolide G (R=CH_3_) (modified from [12]). Dashed lines indicate the cleavage sites resulting in the corresponding fragments.

In this study, SPX with known structures, SPX 1 and a methyl analogue of SPX G, 20-methyl spirolide G (20-Me SPX G), with their CID spectra and fragmentation pattern (Figure 2 and Figure 4, respectively), were used to deduce structural characteristics of the novel spirolides.

### 2.2. Characterization of Novel Spirolides

From the selected reaction monitoring (SRM) scans, the *A. ostenfeldii* strains isolated from The Netherlands, Greenland and Norway collectively revealed ten novel spirolides, which possessed the same masses as previously known spirolides but eluted at different retention times. The pseudo-molecular ions of these unknown spirolides were *m/z* 666 (**2**), *m/z* 696 (**3**), *m/z* 678 (**4**), *m/z* 694 (**5**), *m/z* 708 (**6**), *m/z* 720 (**7**), *m/z* 722 (**8**) and *m/z* 738 (**9**). In addition, another spirolide with pseudo-molecular ion *m/z* 670 (**1**) was identified by precursor ion scans of the characteristic spirolide fragment at *m/z* 164. The accurate masses of the pseudo-molecular ions and the elemental formula of compounds **1–9** were obtained by HR-MS measurements (Table 2, Table 3 and Table 4).

#### 2.2.1. Compound **1**

**Figure 5:**
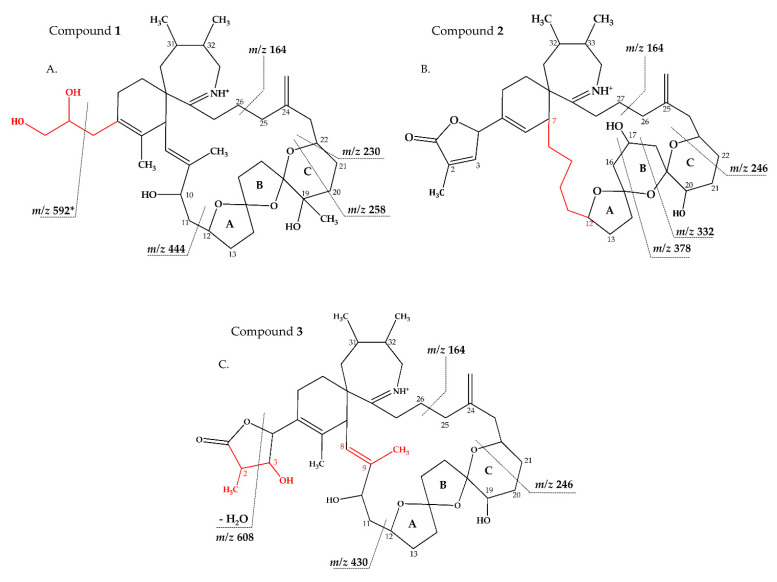
Proposed structures of compounds **1** (**A**) from AON 24 and compounds **2** (**B**) and **3** (**C**) from NX-56-10. Structural parts in red cannot unambiguously be assigned by mass spectrometry. Dashed lines indicate the cleavage sites resulting in the corresponding fragments. * For more information about fragment *m/z* 592, refer to Figure S10.

Strain AON 24, originally isolated from North Sea water of the coast of The Netherlands, predominantly produced SPX 1 (Table 5). We also detected an unknown compound with a CID spectrum inferring an SPX (Figure 1B). Compound **1** had an accurate mass of 670.4678 (C_40_H_64_NO_7_^+^, Table 2 and Table S2), a difference of 22 Da (–C_2_ and +H_2_) in comparison to 13 methyl SPX C (C_42_H_62_NO_7_+). The ring opening via RDA and the subsequent fragmentation of the pseudo-molecular ion produced fragments *m/z* 462 (C_27_H_42_NO_5_^+^), 444 (C_27_H_42_NO_4_^+^), *m/z* 230 (C_16_H_24_N^+^) and *m/z* 164 (C_11_H_24_N^+^), which are also observed for SPX 1, indicating that **1** features an identical triketal ring system and a hydroxyl group at C10 (SPX 1 numeration). In contrast to SPX 1, a fragment was observed at *m/z* 592 (C_38_H_58_NO_4_^+^, Table 2 and Table S2), produced by a neutral loss (NL) of 78 (C_2_H_6_O_3_) for **1**. Assuming a structure identical to SPX 1 starting from C10, this NL can only be explained by a simultaneous loss of water and fragmentation of the butenolide ring ‘part’ of the molecule (one possible fragmentation pathway is shown in Figure 5A). The proposed structure of **1** with a vicinal dihydroxypropyl substituent instead of the butenolide is consistent with the *m/z* 592 fragment formation (Figure 5A and Figure S10), as this fragment can be formed by a nucleophilic attack of one of the hydroxyl groups, which is not possible in the case of the butenolide side chain. Accordingly, the *m/z* 592 fragment usually is not observed in SPX CID spectra. In summary, a structural proposal with a butenolide ring part altered in comparison to SPX 1, featuring a side chain with two hydroxyl groups is the most likely conclusion. However, this structural proposal is based on the assumption that there are no structural changes between C10 and C33 (C-numbering of SPX 1) in comparison to SPX 1 and needs unambiguous confirmation by NMR.

#### 2.2.2. Compound **2**–**3**

Strain NX-56-10, originally isolated from Trondheim Fjord, Norway, produced SPX 1, 20-Methyl SPXG and trace amounts of SPX A, H and I (Table 5). We also detected two unknown SPX in the CID spectra (**2** (Figure 3B) and **3** (Figure 1C)).

Compound **2** had an accurate mass of *m/z* 666.4364 (C_40_H_60_NO_7_^+^, Table 2 and Table S3). The CID spectrum indicated a G-type SPX for compound **2** (Figure 3B), characterized by group 2 (*m/z* 378, 360, 342, 332) and group 3 (*m/z* 246) that were similar to spirolide G (SPCX G). Group 4 (*m/z* 164) cluster was identical to SPX G (Table S1) [12]. The only difference between the SPX G and **2** were found in the group 1 cluster that is characterized by water losses of the parental molecule (*m/z* 666 for **2**, *m/z* 692 for SPX G). The structural difference between both compounds (26 Da, C_2_H_2_) must be located between C1 and C16 (SPX G numeration). However, the low abundant fragment cluster *m/z* 568, 550, 532, 514 (NL 98 Da, Figure 3B and Table 2 and Table S3) that can be explained by the cleavage of the butenolide ring followed by several water losses indicates the presence of the butenolide side chain in **2** that is typical for SPX. The presence of the butenolide ring, however, further narrows down the location of structural differences between SPX G and **2** to C5–C11. As the elemental difference of C_2_H_2_ is impossible to explain by a single modification, several modifications must be present. The most likely assumption is the elimination of the methyl groups at C6 and C9 together with a hydration of a double bond. The only double bond that can be saturated without affecting the RDA macrocycle opening is between C5 and C6. In summary, a structural proposal of 8,9-dihydro-6,9-didesmethyl-SPX G is consistent with the recorded CID spectrum of **2** (Figure 5B). However, this structural proposal is preliminary and needs unambiguous confirmation by NMR.

Compound **3** is characterized by its accurate mass of *m/z* 696.4471 (C_41_H_62_NO_8_^+^, Table 2 and Table S3). The pseudo-molecular ion of **3** (Figure 1C), showed a 4 Da (–C and +O) upshift in comparison to SPX 1 (C_42_H_62_NO_7_^+^, Figure 1A) or an addition of H_2_O in comparison to 13,19-desmethyl SPX C (*m/z* 678). The group 2 fragments of **3** (*m/z* 448, 430, 412; Figure 1C and Table 2 and Table S3) resemble the group 2 fragments of 13,19-didesmethyl SPX C (Table S1) [37] indicating that **3** and 13,19-didesmethyl SPX C share the structural element comprising C12 to C34. This is in agreement with the elemental composition of *m/z* 430 (C_26_H_40_NO_4_^+^) as also observed in the CID spectrum of 13,19-didesmethyl SPX C [15]. Fragment *m/z* 246 (C_17_H_28_N_7_^+^, Table 2) was also observed, indicating no oxygenation in the part of the molecule from C21 to C34 (SPX 1 numeration). The low abundant, but characteristic fragment cluster *m/z* 608, 590, 572 (Table 2 and Table S3) can be explained by a C2-C3 cleavage of the butenolide side chain followed by water losses. This cleavage usually is not observed in SPX, but we propose a 3-hydroxylation, which may cause a destabilization of the butenolide ring and thus explain the characteristic fragmentation by a formation of the *m/z* 608, 590, 572 cluster (Table S3). In summary, the addition of H_2_O is only possible by the saturation of either double bond C2-C3 or C8-C9 or a hydroxylation of the methyl groups at C8 or C9. A structural proposal of 2-hydro-3-hydroxy-13,19-didesmethyl SPX C is consistent with the CID spectrum of **3** (Figure 5C), but the above-mentioned isoforms cannot be ruled out by mass spectrometry.

#### 2.2.3. Compounds 4–9

Strain MX-S-B11, originally isolated from Disko Bay, West Greenland, produced 20-Methyl SPXG, SPX H and I as well as trace amounts of SPX 1 and SPX C (Table 5). We also detected six unknown SPX in the CID spectra (**4–9** Figure 1D,E and Figure 3C–F).

**Figure 6:**
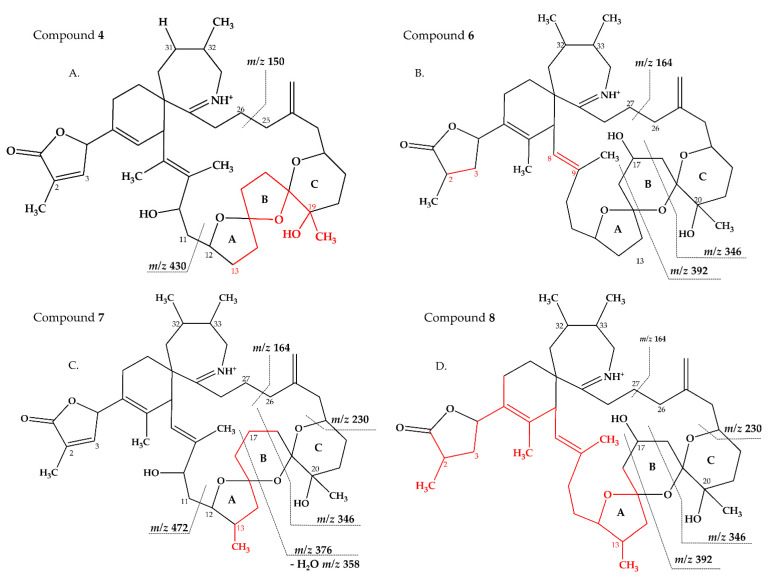
Proposed structures of compounds **4** (**A**), **6** (**B**), **7** (**C**) and **8** (**D**) from MX-S-B11. Structural parts marked in red cannot unambiguously be assigned by mass spectrometry. Dashed lines indicate the cleavage sites resulting in the corresponding fragments.

Compound **4** had an accurate mass of *m/z* 678.4363 (C_41_H_60_NO_7_^+^, Table 3 and S4). This sum formula is identical to 13,19-desmethyl SPX C [37]. The group 2 cluster of **4** (*m/z* 448, 430, 412, 394, Figure 1D and Table 3 and Table S4) was also identical with **3** (Table S3) and 13,19-desmethyl SPX C (Table S1). However, we observed *m/z* 150 (C_10_H_16_N^+^) as the most abundant group 4 ion, which indicated a 14 Da downshift (–CH_2_) in comparison to **3** and 13,19-desmethyl SPX C (*m/z* 164, C_11_H_18_N^+^). The *m/z* 150 fragment can also be observed for SPX A (=31-desmethyl SPX C) (Table S1) [7] which shows no C31 methylation. A structural configuration with a methyl group at C19 (often observed in A-, B-, C- and D-type SPX) and no methyl group of C31 would be in agreement with the observed cluster 4 as well as 2. In summary, the CID spectrum of **4** is consistent with 13-desmethyl SPX A (Figure 6A). Theoretically, another demethylation of C19 instead of C13 is also possible. However, 13-desmethyl variants are more common among SPX.

**Figure 7:**
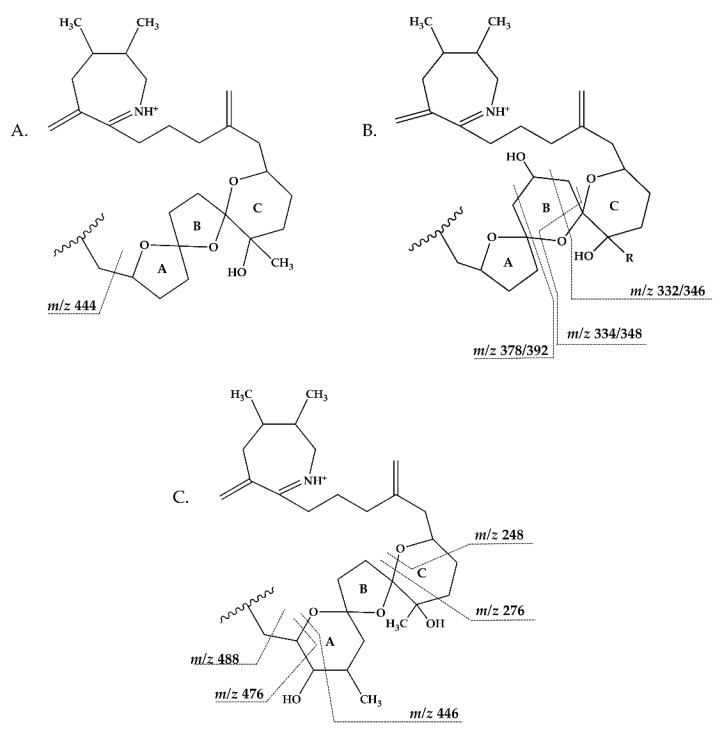
Triketal ring system of **A**: C-type spirolides (modified from [36]) **B**: G-type spirolides (modified from [12]); and **C**: Pinnatoxin E/F (modified from [38]). Dashed lines indicate the cleavage sites resulting in the corresponding fragments.

**Figure 8:**
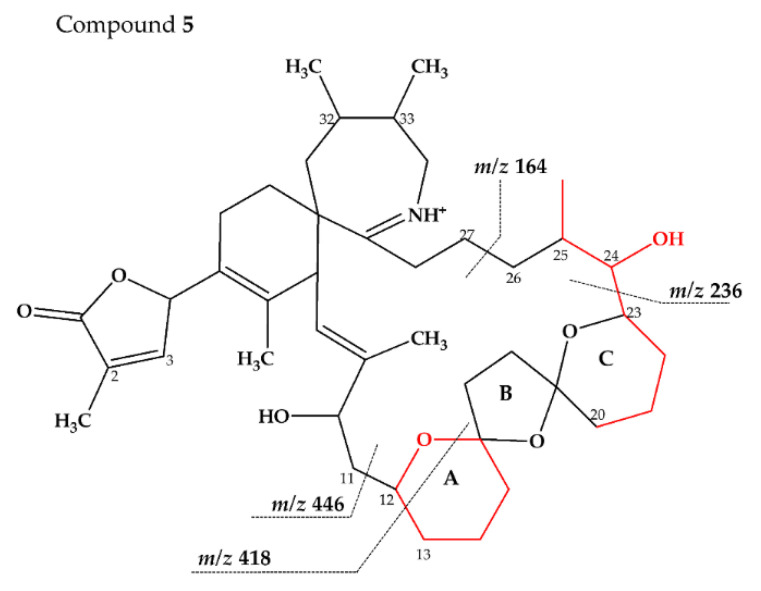
Proposed structure of compound **5** from MX-S-B11. Structural parts marked in red cannot unambiguously be assigned by mass spectrometry. Dashed lines indicate the cleavage sites resulting in the corresponding fragments.

Compound **5** is characterized by its accurate mass of *m/z* 694.4679 with an inferred elemental formula of C_42_H_64_NO_7_^+^ from HR-MS (Table 3 and Table S4). The CID spectrum and HR-MS of **5** (Figure 1E) showed a fragmentation pattern more similar to C-type SPX than G-type SPX. The group 4 fragment (*m/z* 164, C_11_H_18_N^+^) was observed in **5** and SPX 1 (Figure 1E,A, respectively), indicating an identical cyclic imine moiety and C_26_-C_33_ for **5** and SPX 1. Furthermore, the elemental composition of **5** (C_42_H_64_NO_7_^+^) and SPX 1 (C_42_H_62_NO_7_^+^) differed by only two hydrogen atoms. The same was the case for the group 2 fragments C_42_H_64_NO_7_^+^ 464 and 462, respectively, indicating a common neutral loss of 230 Da (C_15_H_18_O_3_) and a conserved structural element of C1–C11 between **5** and SPX 1. Interestingly, unusual ion clusters were observed in group 2 and 3 fragments of **5**. The group 2 cluster showed an additional fragment *m/z* 418 (C_26_H_44_NO_3_^+^) with two corresponding water losses (*m/z* 400 and 382). Fragment *m/z* 418 (Table 3 and Table S4) has not been observed in SPX yet, but could result from the ring opening of ring A (C12–C15) of a putative 5:5:6 ketal ring configuration (Figure 7A). However, an opening of ring A of SPX has not been observed, which suggests a structural difference of the ring A region among all known SPX and **5**. A possible explanation of fragment *m/z* 418 would be a structure that has a 6:5:6 ketal ring configuration (Figure 7C). Considering this hypothetical structural variant, the opening of a six-membered ring A and the bond cleavage between C12–C13 also formed *m/z* 418 (C_26_H_44_NO_3_^+^). An opening of the six-membered ring B of G-type spirolides that have a 5:6:6 triketal ring system (Figure 7B) occurs forming the fragments *m/z* 378 and 392 [12], which are not observed in spirolides with 5:5:6 triketal ring configuration (Figure 2). This leads to the conclusion that **5** may have a 6:5:6 triketal ring system (Figure 8). This hypothesis is supported by the fact that the structurally related pinnatoxins, also with a 6:5:6 triketal ring configuration, in fact show an opening of ring A (Figure 7C). The opening of the six-membered ketal ring A produces fragments *m/z* 446 and 476 for both pinnatoxin E and F [38]. Another characteristic feature in the group 2 fragments is the presence of a high intensity peak at *m/z* 464 (Table 3 and Table S4), which corresponds to the above-mentioned NL of 230 (C_15_H_18_O_2_). It is assumed to be the *m/z* 462 equivalent of SPX 1. In contrast to **5**, *m/z* 462 in SPX 1 [32] like in any other C-type spirolides [11] shows a very low relative abundance, which might be explained by differing configuration of the adjacent ring. This is additional confirmation of the hypothesis that **5** might be composed by a 6:5:6 ring configuration. Another unusual fragment was also observed in group 3, at *m/z* 236 (C_15_H_26_NO^+^, Table 3 and Table S4). This fragment could result from a structure with an addition of a hydroxyl group between C24–C26 and a missing methylene group likely at C25 (Figure 8) in comparison to SPX 1 (Figure 2), resulting in the difference of 6 Da (–C and +H_2_O) in comparison to the *m/z* 230 (C_16_H_24_N^+^) fragment of SPX 1 (Figure 2). The position of the hydroxyl group at C28 can be ruled out for **5**; otherwise, *m/z* 180 should be observed instead of *m/z* 164 in group 4 [8,10]. The additional hydroxyl group at either C24–C26 also requires a missing hydroxyl group likely at C20 (Figure 8) in comparison to SPX 1 (Figure 2).

In summary, we suggest that **5** is a novel spirolide with a 6:5:6 triketal ring system, no methylene and hydroxyl group at C24 and C19 (C-numbering of SPX 1), respectively, and a hydroxyl group between C23–C25 (Figure 8). With the different combinations based from the unique fragments in **5**, it is not possible to propose a full structure with HR-MS data. An alternative structural proposal would include a G-type 5:6:6 ring configuration without a 17-hydroxylation. The absence of a hydroxylation might stabilize and this suppress a fragmentation of ring B (see Compound **7**) and thus explain the absence of the typical G-type fragments. An absence of a hydroxyl group at C10 requires a hydroxylation at a different site, namely of ring A to account for the observed fragments and elemental composition.

Compound **6** had an accurate mass of *m/z* 708.4836 (C_43_H_66_NO_7_^+^, Table 3 and Table S4). The pseudo-molecular ion of **6** (Figure 3C), showed a 2 Da upshift (+H_2_) in comparison to 20-Me SPX G (Figure 3A), while group 2 cluster resulting from the cleavage of ring B (*m/z* 392/346) was identical. The most likely conclusion is that the C2–C3 double bond in the butenolide ring is saturated in **6** (Figure 6B) in comparison to 20-Me SPX G, which also is the case in SPX of the B- and D-type. Alternatively, a saturation could also be located at C8-9, which is less frequently observed in SPX.

Compound **7** had an accurate mass of *m/z* 720.4836 (C_44_H_66_NO_7_^+^, Table 4 and Table S4). Interestingly, **7** (Figure 3D) showed B-ring fragments (*m/z* 376, 358, 346) but also fragments *m/z* 490 and 472. Despite of an overall low intensity in the spectrum, the ratio between *m/z* 490 and 272 was akin to the *m/z* 462 to 444 of SPX 1. Fragment *m/z* 462 in SPX 1 is most likely formed by the charge mediated fragmentation of the C11/12 bond (OH transfer from C10 to C12 and C11/12 cleavage (see 2.1) [32]. Both *m/z* 444 as well as the less abundant *m/z* 462 were only reported for 10-hydroxylated SPX and not for any known G-type SPX [12]. The *m/z* 490/472 (Table 4 and Table S4) cluster of **7** could therefore be equivalent to the *m/z* 462/444 cluster in SPX 1 (Table S1) and point towards a hydroxylation at C10. The fragments of the B-ring cluster showed one hydroxyl group less in comparison to 20-Me SPX G (*m/z* 392, 346, Table S1) indicating no hydroxylation at C18 in **7**. A methyl group between C10 and C18 is also required to satisfy the sum formula, most likely at C13. Contrary to *m/z* 462/444 in the SPX 1 spectrum, *m/z* 490/470 were not most abundant daughter ions (next to *m/z* 164 ion). Instead, the most abundant fragments were observed for the *m/z* 376 cluster. We speculate that the fragmentation of the six-membered B-ring is favored over the hydroxyl attach and subsequent cleavage of the C11–C12 bond. However, this hypothesis needs further investigation as no previous G-type SPX with hydroxyl at C10 is known. In summary, a structure proposal similar to 20-Me SPX G but with a hydroxyl group at C10, no hydroxyl at C18 and methyl group at C13 (Figure 6C) would satisfy all observed fragmentation characteristics.

Compound **8** had an accurate mass of *m/z* 722.4994 (C_44_H_68_NO_7_^+^, Table 4 and Table S4). The CID spectrum of **8** (Figure 3E) displayed an identical fragment pattern as 20-Me SPX G (Table S1) with the exception of an upshift of 16 Da (+CH_4_) in the group 1 fragments in **8**. The upshifted NL resulting from the cleavage between C16 and C17 (*m/z* 314 in 20-Me SPX G, *m/z* 330 in **8**) indicates a methyl group between C3 and C16 and a saturation at either C8-C9 or C2-C3 of **8**. In summary, the proposed structure of 8 is made up of a methyl analogue of 20-Me SPX G (Figure 6D).

**Figure 9:**
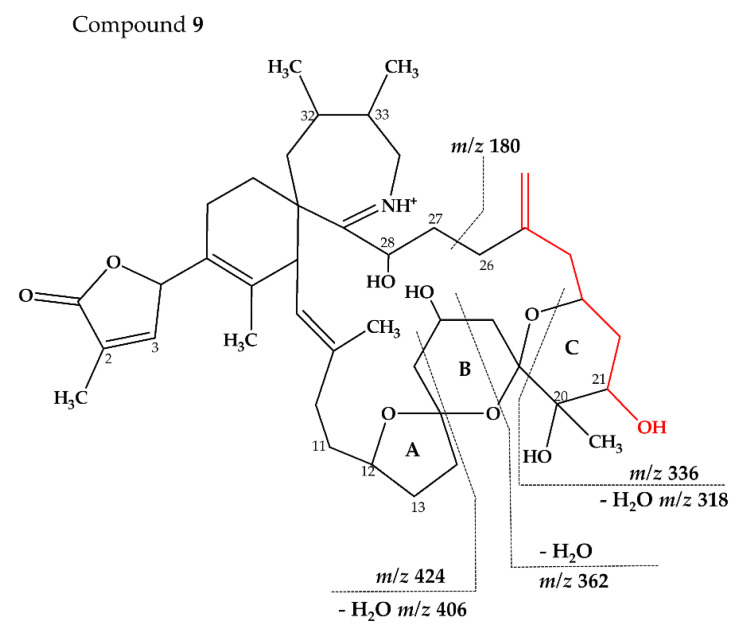
Proposed structures of compounds **9** from MX-S-B11. Structural parts marked in red cannot unambiguously be assigned by mass spectrometry. Dashed lines indicate the cleavage sites resulting in the corresponding fragments.

Compound **9** had an accurate mass of m/z 738.4579 (C_43_H_64_NO_9_^+^, Table 4 and Table S4). The CID spectrum of **9** (Figure 3F) showed fragment *m/z* 180 that is found in 27-hydroxy SPX analogues [8,10] (corresponding to 28-hydroxy G-type SPX). At first sight, the intensities did neither resemble a G-type nor a C-type SPX fragmentation. However, low intensity fragments indicated a G-type spirolide with an upshift of 16 (O_2_) in the B-ring fragment (*m/z* 424, Table 4 and Table S4) indicating two more hydroxyl groups in comparison to 20-Me SPX G (*m/z* 392, 346, Table S4). A fragment at *m/z* 362 was also observed which could correspond to the B-ring cleavage after a loss of water elsewhere. In contrast to 20-Me SPX G, the most intense fragments of this cluster were observed for the additional loss of H_2_O (*m/z* 406, 362, Table 4 and Table S4). A similar observation was made for the most abundant fragment ion (*m/z* 318, Table 4 and Table S4) of the spectrum, which indicated one loss of water from *m/z* 336 (C_20_H_34_NO_3_^+^). We suggest, that the unusually high abundance of this C-ring fragment is due to an additional hydroxyl group at C21, which destabilize the ring and favors the *m/z* 336/318/300 over the B-ring cluster formation. In summary, the mass spectrum suggests a structure with two more hydroxyl groups between C21 and C28 in comparison to 20-Me SPX G, with one hydroxyl most likely at C28 and the other at C21 (Figure 9).

### 2.3. Structural Variability of Spirolides

This mass spectrometry-based study confirms previous studies which also indicate a high structural diversity of spirolide congeners produced by *Alexandrium ostenfeldii* [7,8,10,12,13,19,21,22,25]. Moreover, this study provides first evidence for a new SPX subclass with a novel triketal ring system configuration. All other previously described SPX only differ in ring B configuration (five or six carbon atoms for C-type or G-type SPX, respectively) and have a similar configuration of their ring A and C [39]. The only reported exceptions are dispiroketal H/I-type SPX that do not have a ring C, but a 5:6 configuration, instead. Most SPX of this study belong to either C-type or G-type SPX with 5:5:6 and 5:6:6 triketal ring system configuration. However, mass spectral data of **5** suggest a six-membered ketal ring A (6:5:6 configuration). The postulated 6:5:6 triketal ring system of **5** was previously only observed in pinnatoxins, which otherwise feature a seven-membered ether ring instead of a butanolide side chain. Pinnatoxins and spirolides resemble each other in the last 2/3rds of the chain covering the ketal ring system (Figure 7) indicating that they may share common nascent polyketide chain in the biosynthesis of this part of the molecule [39]. While both toxins contain the same cyclic imine moiety (represented by *m/z* 164), they produce different fragmentation patterns and no compound with pinnatoxin-type fragmentation was detected in the present study nor is any reported in previous studies of *A. ostenfeldii*. Similarly, spirolides compounds are not detected in *Vulcanodinium rugosum* that produces pinnatoxins [40]. On the other hand, the remaining part of SPX including the butenolide side chain is identical to that of gymnodimines [39], again suggesting that identical biosynthetic steps are involved in the synthesis of both compounds. Contrary to pinnatoxins, gymnodimines, initially identified in *Karenia selliformis* [41], haven’t been reported to co-occur with spirolides in *A. ostenfeldii* [20,21,39].

Aside from the fundamental change in the triketal ring system, other proposed structural differences of compound **1**–**9** include the degree of saturation and number of attached methyl or hydroxyl groups. These structural differences can result from small differences in ketoreduction (KR), dehydration (DH) and enoyl reduction (ER). The presence of double bonds, for example, results from omitting the ER process, while the presence of hydroxyl group results from omission of the DH process.

SPX variability in *Alexandrium ostenfeldii* is obvious between different strains often having different SPX profiles [7,8,10,12,13,19,21,22,25]. The toxin profile of a given strain is thought to be genetically fixed and, consistent with that, the relative spirolide composition of the strains studied here remained constant at least over a period of ten months in culture (data not shown). It is thus valid to discuss toxin profile differences among strains (Table 5). While all three strains share the presence of SPX 1 and 20-methyl SPX G, the relative contribution of each single known compound may vary significantly. For example, SPX 1 is the major compound in strain AON 24 (99.90%) but only a minor compound in strain NX-56-10 (6.37%) and MX-S-B11 (0.73%). Notably, 13,19-didesmethyl spirolide C was the major component of NX-56-10 (88.85%). All new compounds are minor components in terms of relative percentage, with the exception of **6** that contributes 18.51% in strain MX-S-B11. Moreover, certain SPX types only were produced in a single strain (Table 5). The high structural variability of secondary compounds of microalgae such as phycotoxins is not uncommon and also has been observed in other species than *A. ostenfeldii*, for example in *Protoceratium reticulatum* with more than 90 yessotoxin variants [42] or in *Prymnesium parvum* with more than 50 prymnesin variants [43]. A possible explanation for a high structural variability of SPX could be that some variants might be biosynthetic precursors of others. However, the fact that different variants are found as major compounds in several strains argues against this hypothesis. Another, more likely explanation may be that chemical variability may be an evolutionary trait in order to improve chemical plasticity and thereby better allow species to adapt to environmental changes. However, since the biological function of most phycotoxins including SPX still remain unknown, this hypothesis still needs confirmation and certainly more research is needed to address the biological functions of microalgal secondary metabolites.

## 3. Materials and Methods

### 3.1. Culture Conditions and Cell Harvest

**Figure 10:**
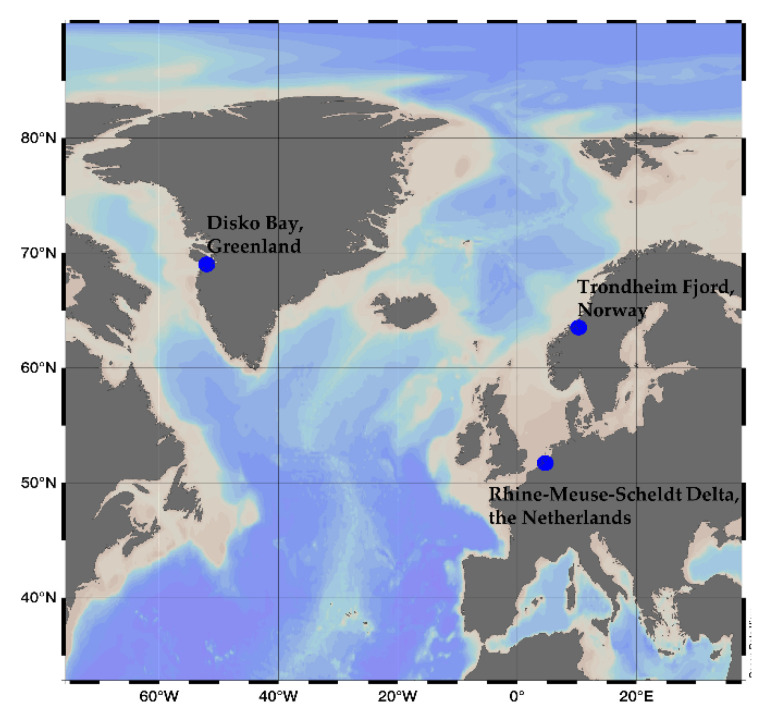
Sampling locations of the *Alexandrium ostenfeldii* strains used in this study.

The *Alexandrium ostenfeldii* strains analyzed in this study were collected in The Netherlands, Greenland and Norway. The clonal strains AON 24 from The Netherlands was collected in the Rhine-Meuse-Scheldt Delta, The Netherlands (51.73° N, 4.72° E), while MX-S-B11 and NX-56-10 were collected in Disko Bay, Greenland (69.03° N, 52.04° W) and Trondheim Fjord, Norway (63.52° N, 10.28° E), respectively (Figure 10). Strains MX-S-B11 and NX-56-10 were morphologically identified by plate pattern analyis using epifluorescence microscopy, and species identification was confirmed by rDNA large-subunit (LSU) sequence comparison (Tillmann, unpublished), and strain AON 24 was identified by microsatellite genotyping [44] of loci published in Nagai et al. [45] *A. ostenfeldii* strains were grown in 70 mL plastic culture flasks with filtered K medium (Keller, 1987) adjusted to pH 8 at salinity 30 (MX-S-B11 and NX-56-10) or 10 (AON 24) and were kept under a 16:8 light-dark cycle at 15 °C with 50 μmol m^−2^ s^−1^ photon flux density. Cell density was determined by fixing 1 mL of microlagal culture in a counting chamber with Lugol’s iodine solution and counting the cells under 100× magnification with an Axiovert 40C optical microscope (Zeiss, Göttingen, Germany). Cultures that reached cell densities of >1500 cells mL^−1^ were harvested by centrifugation of 50 mL culture at 3,220 × g for 10 min (Eppendorf 5810R, Hamburg, Germany). The cell pellets were transferred to 1 mL microtubes, centrifuged at 16,000× *g* for 5 min (Eppendorf 5415R, Hamburg, Germany) and stored at −20 °C until extraction.

### 3.2. Spirolide Extraction

Cell pellets were suspended in 500 μL methanol and mixed in a vortex mixer (Heidolph, Schwabach, Germany). The suspension was transferred into a spin-filter (pore-size 0.45 mm, Millipore Ultrafree, Eschborn, Germany) and centrifuged at 16,000× *g* for 15 min (Eppendorf 5415R, Hamburg, Germany). The filtrate was transferred into LC vials for mass spectrometric measurement.

### 3.3. Parameters of Liquid Chromatography-Tandem Mass Spectrometry (LC-MS/MS)

A triple-quadrupole mass spectrometer (API 4000 Q Trap, Sciex, Darmstadt, Germany) with a Turbo V ion source coupled to a 1100 LC liquid chromatograph (Agilent, Waldbronn, Germany) was used in the detection of novel spirolides.

#### 3.3.1. Liquid Chromatography (LC)

The LC component, composed of solvent reservoir, in-line degasser (G1379A), binary pump (G1311A), refrigerated autosampler (G1329A/G1330B) and a temperature-controlled column oven (G1316A) was employed for the toxin separation which was conducted using an analytical C8 reverse phase column (50 mm × 2 mm) packed with 3 μm Hypersil BDS 120 Å (Phenomenex, Aschaffenburg, Germany) and thermostated at 20 °C. A gradient elution was then performed with water and methanol/water (95:5 v/v) as eluent A and B, respectively, and a buffer system made of ammonium formate and formic acid was added to both eluents with final eluent concentrations of 2.0 mM and 50 mM, respectively. A flow rate of 0.2 mL min^−1^ was used to generate a gradient flow of 5% eluent B during the initial condition, a linear gradient to 100% B up to 10 min after injection, an isocratic elution for 20 min, 5% B for 1 min and finally 9 min for column equilibration. The total run time was 30 min.

#### 3.3.2. Scan Modes in Tandem Mass Spectrometry (MS/MS)

Different tandem mass spectrometry (MS/MS) scan modes were used in this study: Selected reaction monitoring (SRM), precursor ion scan and enhanced product ion (EPI) scan. SRM is a highly selective quantitative target analyte scan but requires prior information of the precursor and product ions of the compound of interest. In SRM, the precursor ion was selected in the first quadrupole (Q1), fragmented in the collision cell by collision-induced dissociation (CID) and the product ion was selected in the third quadrupole (Q3) for detection. The following mass spectrometric parameters were applied in SRM: Curtain gas: 20 psi, CAD (collision activated dissociation) gas: Medium, ion-spray voltage: 5500 V, temperature: 650 °C, nebulizer gas: 40 psi, auxiliary gas: 70 psi, interface heater: On, declustering potential: 121 V, entrance potential: 10 V, exit potential: 22 V.

In order to identify novel compounds, samples were subjected to precursor ion scan of *m/z* 164, which is characteristic to spirolides. Precursor ions were scanned in Q1 with a mass range set at 500-800 Da, fragmented in the collision cell and the product ion, *m/z* 164, was selected in Q3. The following mass spectrometric parameters were applied in the precursor ion scan: Curtain gas: 20 psi, CAD (collision activated dissociation) gas: High, ion-spray voltage: 5500 V, temperature: 650 °C, nebulizer gas: 40 psi, auxiliary gas: 70 psi, interface heater: On, declustering potential: 121 V.

Finally, the compounds that were identified as potential spirolides in the precursor ion scan were subjected to enhanced product ion (EPI) scan to obtain CID spectra. In the EPI scan, the precursor ion or the pseudo-molecular ion ([M + H]^+^) was scanned in Q1 and fragmented in Q2. As the fragmented ions exit from Q2, they enter in Q3 that accumulates the fragment ions by subjecting them to a highly sensitive ion trap mass scan. The following mass spectrometric parameters were applied in EPI: Curtain gas: 20 psi, CAD (collision activated dissociation) gas: High, ion-spray voltage: 5500 V, temperature: 650 °C, nebulizer gas: 40 psi, auxiliary gas: 70 psi, interface heater: On, declustering potential: 121 V, entrance potential: 10 V, exit potential: 22 V.

### 3.4. Analyses of Spirolides by High Resolution Tandem Mass Spectrometry (HR-MS/MS)

High-resolution mass measurement and fragmentation spectra of novel spirolides were gathered using UHPLC system coupled to a hybrid quadrupole mass spectrometer (Vanquish UHPLC, Q Exactive Plus HR-MS, both Thermo Fisher Scientific, Schwerte, Germany) with a heated electrospray ionization source. Separation was performed on a C18 column (C18 BEH, 100 × 2 mm, 1.7 µm particle size, ACQUITY (Waters, Eschborn, Germany) equipped with guard column) with a column oven set to 32 °C. Samples were eluted using solvent A (H_2_O + 10 mM ammonium formate and 0.1% formic acid) and solvent B (ACN + 10 mM ammonium formate and 0.1% formic acid), which followed a stepwise gradient of A:B (90:10) to 100% B at a flow rate of 0.55 mL min^−1^. In addition, the elution of the first 0.6 min of each run were discarded to waste to minimize the presence of salt deposits. All measurements were performed with the capillary temperature set to 266 °C, the auxiliary gas heater to 400 °C, the spray voltage of 3.5 kV, the sheath gas flow of 51 and the auxiliary gas rate of 18. Positive Ion Calibration Solution (Pierce, Thermo Fisher) was used for the calibration of the instrument.

Assignment of elemental formulas were done from HRMS/MS spectra acquired in data independent acquisition (DIA) mode. DIA spectra were acquired with a resolution (RES) of 280000 FWHM (*m/z* 200), automatic gain control (AGC) of 5 × 10^5^, isolation range of 1.0–3.0 *m/z* and normalized collision energy of 30 utilizing an inclusion list (*m/z* 666.44, 670.44, 678.44, 694.47, 696.48, 708.48, 720.48, 722.50 and 738.46). The exact mass of the pseudo-molecular ion of compound 9 (*m/z* 708) was taken from a full scan (RES = 280000 (*m/z* 200), AGC = 2 × 10^5^, MaxIT = 50, SR = *m/z* 6500 to 750). Analytes with >3 point per peak were successfully detected in all experiments. The assignment for the molecular formulas of the [M + H]^+^ ions were set within the acceptable mass tolerance range of the instrument’s error (<1ppm). After the application of the nitrogen rule as well as some general considerations (e.g., molecular formula or carbon number of the daughter ions in comparison to the formula of the pseudo-molecular ion), generally only one theoretical elemental formula (C, H, O, N atoms only) corresponded to the measured *m/z* within 1 ppm.

## 4. Conclusions

The mass spectrometry-based structural characterization used in this study resulted in the proposal of eight novel SPX that have either a 5:5:6 or 5:6:6 triketal ring configuration and one SPX that may have a 6:5:6 ring configuration otherwise known from closely related pinnatoxins. However, future studies by NMR will be needed to confirm the structural features derived from mass spectrometric analyses of this work. Even though each strain seems to have a dominance of one or the other triketal ring system, these ring systems are not exclusive of one strain, but different ring systems are usually present indicating a high variability of biosynthetic pathways in this species. As the genes encoding for SPX biosynthesis have not been described yet, unravelling of the involved PKS genes in SPX biosynthesis will add valuable information for the understanding of the expression of this class of secondary metabolites and their putative physiological and/or ecological functions. In addition, toxicity of the novel SPX needs to be assessed to get more insights into structure-toxicity relationships. Structurally related pinnatoxins have an increased oral toxicity compared to SPX (**5**), which might be due to their 6:5:6 ketal ring configuration, which also may be the case for SPX with a 6:5:6 ketal ring configuration. Less is known of other structural elements of SPX such as 10-hydroxylation, which is a commonly observed feature of C-type SPX, but also in **7**, which is the first report on a 10-hydroxy G-type SPX, and of the butenolide side chain, which is shared by almost all SPX and also gymnodimines. In this sense, toxicity testing of the novel compounds, especially of **1** (truncated butenolide side chain), **5** (6:5:6 ketal ring configuration) and **7** (10-hydroxy G-type SPX), would be of special interest. However, for both NMR analysis and toxicity testing, relatively high amounts of purified compounds in the µg range are necessary, which currently hampers progress in this area.

## Figures and Tables

**Table 1:** Structurally elucidated spirolides from *Alexandrium ostenfeldii* and their corresponding mass transition.

Spirolide	R_1_	R_2_	R_3_	R_4_	R_5_	R_6_	Δ^C2,3^	Mass Transition	Structure	Reference
A	H	CH_3_	CH_3_	H	H	OH	+	692 > 150	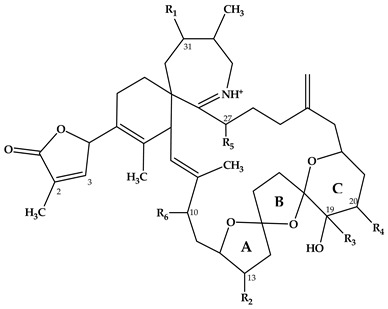	[7]
B	H	CH_3_	CH_3_	H	H	OH	-	694 > 150		[3,4]
C	CH_3_	CH_3_	CH_3_	H	H	OH	+	706 > 164		[7]
13-desMethyl C	CH_3_	H	CH_3_	H	H	OH	+	692 > 164		[7]
13,19-didesMethyl C	CH_3_	H	H	H	H	OH	+	678 > 164		[9,15]
20-Hydroxy-13,19-didesMethyl C	CH_3_	H	H	OH	H	OH	+	694 > 164		[11]
27-Hydroxy-13-desMethyl C	CH_3_	H	CH_3_	H	OH	OH	+	708 > 180		[10]
27-Hydroxy-13,19-didesMethyl C	CH_3_	H	H	H	OH	OH	+	694 > 180		[10]
27-Oxo-13,19-didesMethyl C	CH_3_	H	H	H	O	OH	+	692 > 178		[10]
D	CH_3_	CH_3_	CH_3_	H	H	OH	-	708 > 164		[3]
13-desMethyl D	CH_3_	H	CH_3_	H	H	OH	-	694 > 164		[8]
20-Hydroxy-13,19-didesMethyl D	CH_3_	H	H	OH	H	OH	-	696 > 164		[11]
G	CH_3_	H	H	H	H	H	+	692 > 164	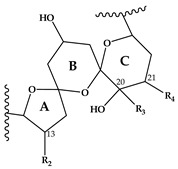	[12]
20-Methyl G	CH_3_	H	CH_3_	H	H	H	+	706 > 164		[12]
H	CH_3_	H	CH_3_	H	H	OH	+	650 > 164	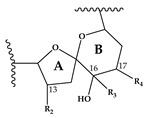	[13]
I	CH_3_	H	CH_3_	H	H	OH	-	652 > 164		[13]

**Table 2:** Measured and calculated accurate masses of the key product ions of compounds **1** from AON 24 and NX-56-10 obtained by HR-MS.

Strain	Compound	Elemental Formula	Measured	Calculated	Δ/ppm
AON 24 (The Netherlands)	**1** (670 > 164)	C_40_H_64_NO_7_^+^	670.4678	670.4677	0.07
		C_38_H_58_NO_4_^+^	592.4363	592.4360	0.51
		C_27_H_44_NO_5_^+^	462.3219	462.3214	1.01
		C_27_H_42_NO_4_^+^	444.3111	444.3108	0.66
		C_18_H_28_N^+^	258.2217	258.2216	0.21
		C_16_H_24_N^+^	230.1904	230.1903	0.32
		C_11_H_18_N^+^	164.1435	164.1434	0.81
NX-56-10 (Norway)	**2** (666 > 164)	C_40_H_60_NO_7_^+^	666.4365	666.4364	0.14
		C_35_H_50_NO_3_^+^	532.3787	532.3785	0.38
		C_22_H_36_NO_4_^+^	378.2640	378.2639	0.32
		C_22_H_34_NO_3_^+^	360.2533	360.2533	−0.07
		C_21_H_34_NO_2_^+^	332.2584	332.2584	−0.13
		C_17_H_28_N^+^	246.2216	246.2216	0.09
		C_11_H_18_N^+^	164.1435	164.1434	0.62
	**3** (696 > 164)	C_41_H_62_NO_8_^+^	696.4471	696.4470	0.13
		C_38_H_54_NO_3_^+^	572.4100	572.4098	0.37
		C_26_H_42_NO_5_^+^	448.3060	448.3057	0.56
		C_26_H_40_NO_4_^+^	430.2954	430.2952	0.52
		C_17_H_28_N^+^	246.2217	246.2216	0.34
		C_11_H_18_N^+^	164.1435	164.1434	0.90

**Table 3:** Measured and calculated accurate masses of the key product ions of compounds **4**–**6** from MX-S-B11 obtained by HR-MS.

Strain	Compound	Elemental Formula	Measured	Calculated	Δ/ppm
MX-S-B11 (Greenland)	**4** (678 > 150)	C_41_H_60_NO_7_^+^	678.4363	678.4364	−0.22
		C_26_H_42_NO_5_^+^	448.3059	448.3057	0.42
		C_26_H_40_NO_4_^+^	430.2953	430.2952	0.38
		C_17_H_26_N^+^	244.2061	244.2060	0.32
		C_10_H_16_N	150.1278	150.1277	0.64
	**5** (694 > 164)	C_42_H_64_NO_7_^+^	694.4679	694.4677	0.24
		C_29_H_48_NO_4_^+^	474.3581	474.3578	0.65
		C_27_H_46_NO_5_^+^	464.3374	464.3370	0.76
		C_26_H_44_NO_3_^+^	418.3318	418.3316	0.59
		C_15_H_26_NO^+^	236.2010	236.2009	0.35
		C_11_H_18_N^+^	164.1435	164.1434	0.90
	**6** (708 > 164)	C_43_H_66_NO_7_^+^	708.4836 *	708.4834	1.06
		C_23_H_38_NO_4_^+^	392.2798	392.2795	0.56
		C_22_H_36_NO_2_^+^	346.2744	346.2741	0.85
		C_11_H_18_N^+^	164.1439	164.1434	−0.08

*m/z taken from full scan (see comment Figure S6).

**Table 4:** Measured and calculated accurate masses of the key product ions of compounds **7–9** from MX-S-B11 obtained by HR-MS.

Strain	Compound	Elemental Formula	Measured	Calculated	Δ/ppm
MX-S-B11 (Greenland)	**7** (720 > 164)	C_44_H_66_NO_7_^+^	720.4836	720.4834	0.37
		C_29_H_46_NO_5_^+^	490.3530	490.3527	0.60
		C_29_H_44_NO_4_^+^	472.3425	472.3421	0.70
		C_23_H_38_NO_3_^+^	376.2849	376.2846	0.77
		C_23_H_36_NO_2_^+^	358.2741	358.2741	0.05
		C_22_H_38_NO_2_^+^	346.2743	346.2741	0.21
		C_16_H_24_N^+^	230.1905	230.1903	0.58
		C_11_H_18_N^+^	164.1435	164.1434	0.72
	**8** (722 > 164)	C_44_H_68_NO_7_^+^	722.4994	722.4990	0.50
		C_23_H_38_NO_4_^+^	392.2796	392.2795	0.17
		C_23_H_36_NO_3_^+^	374.2691	374.2690	0.28
		C_22_H_38_NO_3_^+^	364.2691	364.2846	−0.12
		C_22_H_36_NO_2_^+^	346.2741	346.2741	0.24
		C_18_H_28_N^+^	258.2217	258.2216	0.32
		C_16_H_24_N^+^	230.1904	230.1903	0.45
		C_11_H_18_N^+^	164.1435	164.1434	0.72
	**9** (738–180)	C_43_H_64_NO_9_^+^	738.4579	738.4576	0.44
		C_23_H_38_NO_6_^+^	424.2697	424.2694	0.68
		C_22_H_36_NO_3_^+^	362.2693	362.2690	0.79
		C_20_H_32_NO_3_^+^	336.2537	336.2533	1.10
		C_20_H_32_NO_2_^+^	318.2429	318.2428	0.42
		C_15_H_26_NO^+^	236.2010	236.2009	0.67
		C_11_H_18_NO^+^	180.1384	180.1383	0.59

**Table 5:** Cell quota and relative SPX content of *A. ostenfeldii* strains from The Netherlands (AON 24), Norway (NX-56-10) and Greenland (MX-S-B11). Nd refers to *not detected* and LOD refers to the *limit of detection*.

Spirolide	MX-S-B11 (Greenland)		NX-56-10 (Norway)		AON 24 (The Netherlands)		Ketal Ring System
	Cell Quota (fg cell^−1^)	Relative Content (%)	Cell Quota (fg cell^−1^)	Relative Content (%)	Cell Quota (fg cell^−1^)	Relative Content (%)	(A:B:C)
***Known***							
13-desMethyl spirolide C	34.5	0.73	146.1	6.37	740.3	99.90	5:5:6
20-Methyl spirolide G	2086.4	44.41	5.1	0.22	0.4	0.05	5:6:6
Spirolide A	384.3	8.18	0.3	0.01	<LOD (0.06)	Nd	5:5:6
Spirolide C	33.9	0.72	0.1	0.01	0.1	0.02	5:5:6
13,19-didesMethyl spirolide C	<LOD (0.16)	Nd	2037.9	88.85	<LOD (0.06)	Nd	5:5:6
Spirolide H	257.3	5.48	0.4	0.02	<LOD (0.06)	Nd	5:6
Spirolide I	158.4	3.37	0.3	0.01	<LOD (0.06)	Nd	5:6
***Unknown***							
Compound **1**	<LOD (0.16)	Nd	<LOD (0.07)	Nd	0.2	0.03	5:5:6
Compound **2**	<LOD (0.16)	Nd	81.0	3.53	<LOD (0.06)	Nd	5:6:6
Compound **3**	<LOD (0.16)	Nd	22.3	0.97	<LOD (0.06)	Nd	5:5:6
Compound **4**	19.6	0.42	<LOD (0.07)	Nd	<LOD (0.06)	Nd	5:5:6
Compound **5**	233.7	4.98	<LOD (0.07)	Nd	<LOD (0.06)	Nd	6:5:6
Compound **6**	883.2	18.80	<LOD (0.07)	Nd	<LOD (0.06)	Nd	5:6:6
Compound **7**	423.5	9.02	<LOD (0.07)	Nd	<LOD (0.06)	Nd	5:6:6
Compound **8**	182.0	3.87	<LOD (0.07)	Nd	<LOD (0.06)	Nd	5:6:6
Compound **9**	0.8	0.02	<LOD (0.07)	Nd	<LOD (0.06)	Nd	5:6:6

## Supplementary Materials

The following are available online at https://www.mdpi.com/1660-3397/18/10/505/s1, Figure S1: HRMS/MS spectrum of compound 1 (670>164) from AON 24, Figure S2: HRMS/MS spectrum, Figure S3: HRMS/MS spectrum of compound 3 (696>164) from NX-56-10, Figure S4: HRMS/MS spectrum of compound 4 (678>150) from MX-S-B11, Figure S5: HRMS/MS spectrum of compound 5 (694>164) from MX-S-B11, Figure S6: HRMS/MS spectrum of compound 6 (708>164) from MX-S-B11, Figure S7: HRMS/MS spectrum of compound 7 (720>164) from MX-S-B11, Figure S8: HRMS/MS spectrum of compound 8 (722>164) from MX-S-B11, Figure S9: HRMS/MS spectrum of compound 9 (738>180) from MX-S-B11, Figure S10: Proposed fragmentation pattern of *m/z* 592 from compound 1 (670>164), Table S1: Extended fragment list from known spirolides, Table S2: Extended HRMS/MS fragment list of compounds 1 from AON 24, Table S3: Extended HRMS/MS fragment list of compounds 2 and 3 from NX-56-10, Table S4: Extended HRMS/MS fragment list of compounds 4-9 from MX-S-B11.
