# Supplementary material for: Mass Spectrometry-Based Characterization of New Spirolides from Alexandrium ostenfeldii (Dinophyceae)
Source: Mar Drugs. 2020 Oct 2;18(10):505. doi: 10.3390/md18100505 (PMC7599687; doi:10.3390/md18100505)
Supplement: Supplementary file 1 [file marinedrugs-18-00505-s001.pdf]

Supporting Information

Mass Spectrometry-Based Characterization of New Spirolides from *Alexandrium ostenfeldii* (Dinophyceae)

Figure S1. HRMS/MS spectrum of compound **1** (670>164) from AON 24 ....S2

Figure S2. HRMS/MS spectrum of compound **2** (666>164) from NX-56-10. ....S3

Figure S3. HRMS/MS spectrum of compound **3** (696>164) from NX-56-10 ....S3

Figure S4. HRMS/MS spectrum of compound **4** (678>150) from MX-S-B11. ....S4

Figure S5. HRMS/MS spectrum of compound **5** (694>164) from MX-S-B11 ....S4

Figure S6. HRMS/MS spectrum of compound **6** (708>164) from MX-S-B11 ....S5

Figure S7. HRMS/MS spectrum of compound **7** (720>164) from MX-S-B11 ....S6

Figure S8. HRMS/MS spectrum of compound **8** (722>164) from MX-S-B11 ....S6

Figure S9. HRMS/MS spectrum of compound **9** (738>180) from MX-S-B11 ....S7

Figure S10. Proposed fragmentation pattern of *m/z* 592 from compound **1** (670>164) S8

Table S1. Extended fragment list from known spirolides S9

Table S2. Extended HRMS/MS fragment list of compounds **1** from AON 24 ....S10

Table S3. Extended HRMS/MS fragment list of compounds **2** and **3** from NX-56-10............................S11

Table S4. Extended HRMS/MS fragment list of compounds **4-9** from MX-S-B11 ....S12

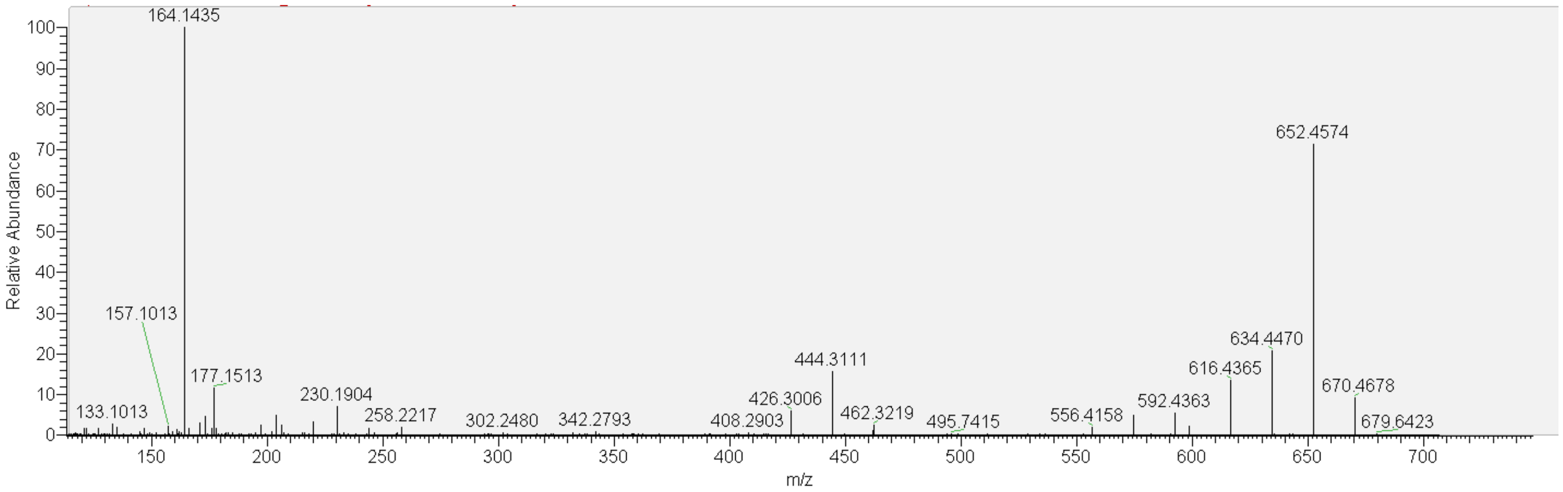

Figure S1. HRMS/MS spectrum of compound **1** (670>164) from AON 24.

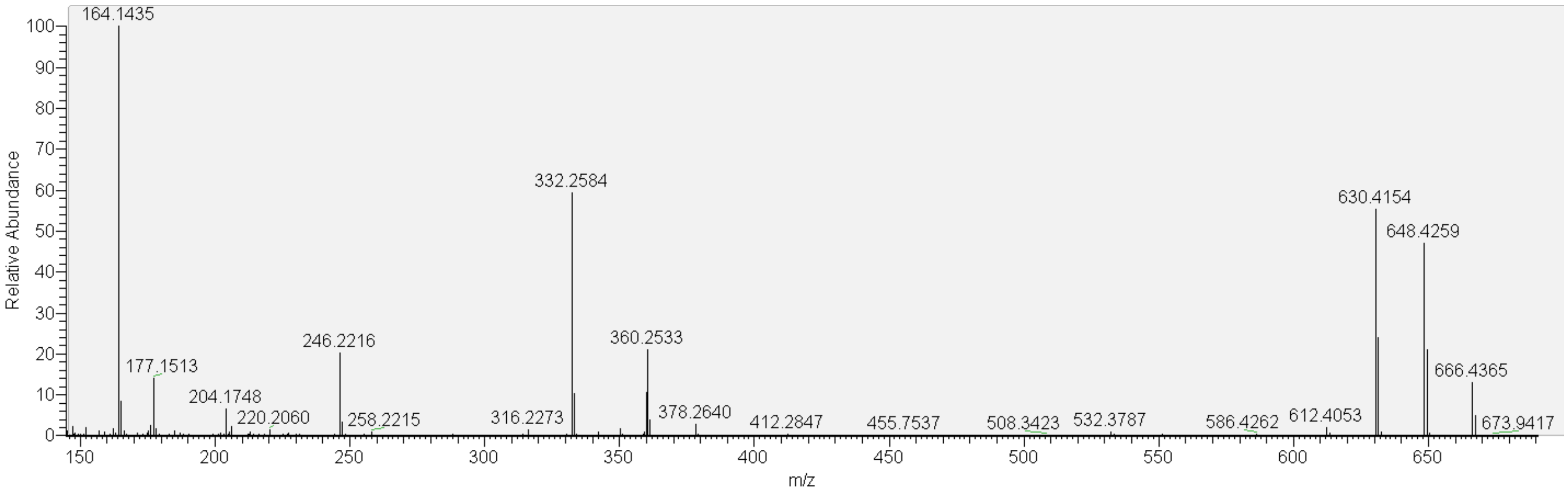

Figure S2. HRMS/MS spectrum of compound **2 (**666>164) from NX-56-10.

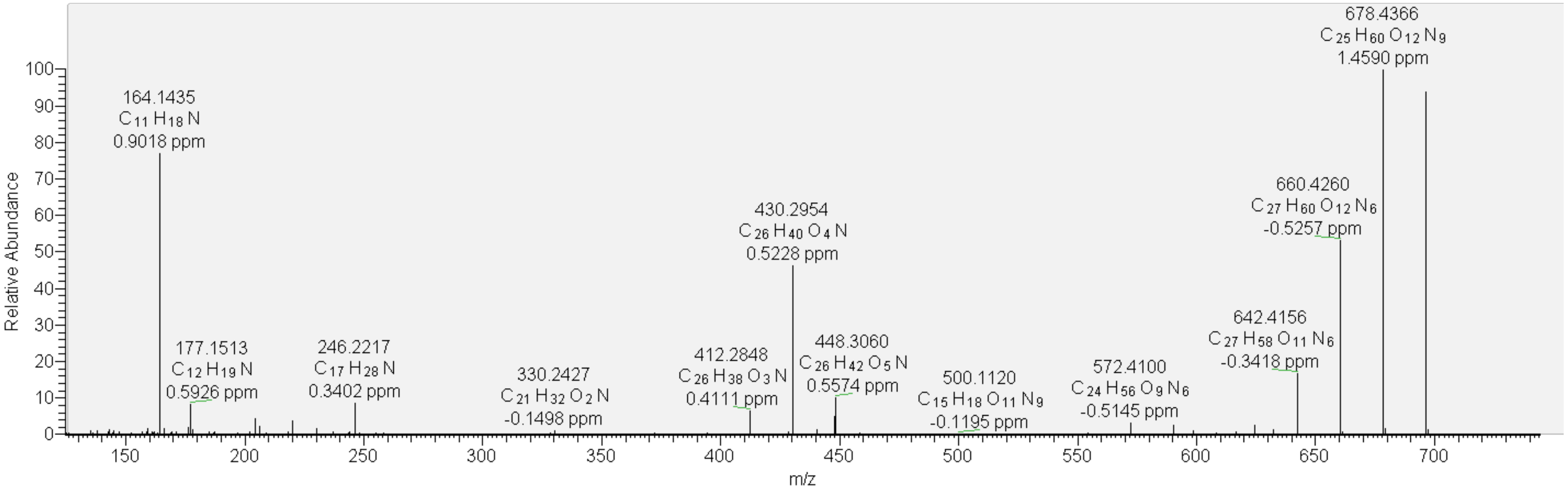

Figure S3. HRMS/MS of compound **3 (**696>164) from NX-56-10.

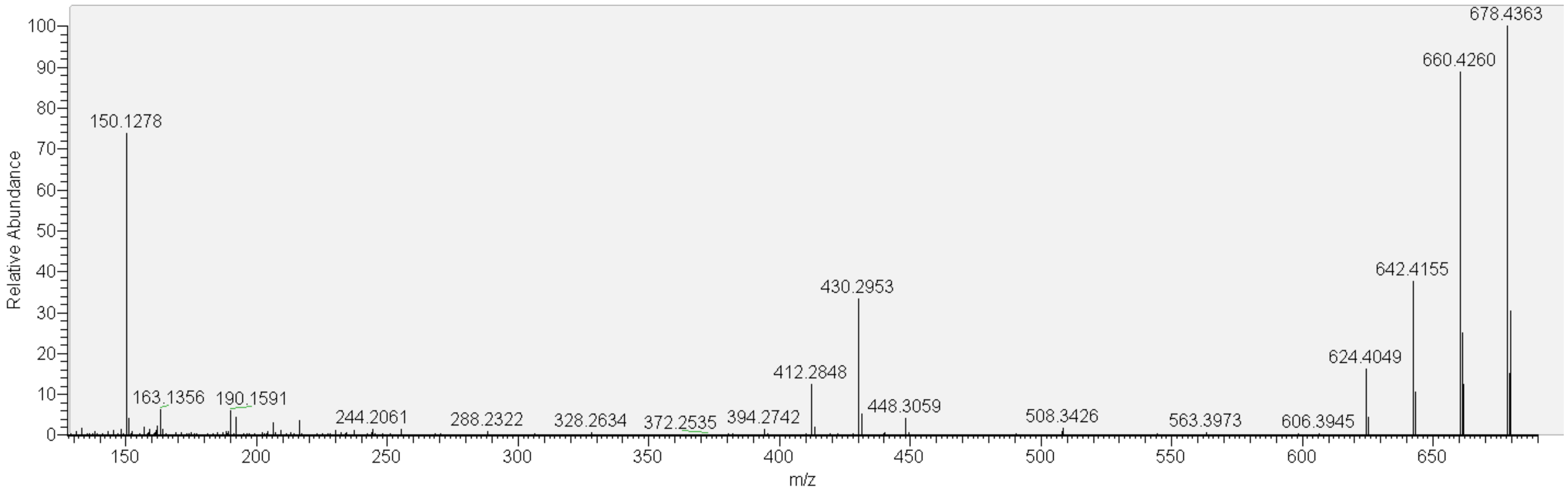

Figure S4. HRMS/MS spectrum of compound **4** (678>150) from MX-S-B11.

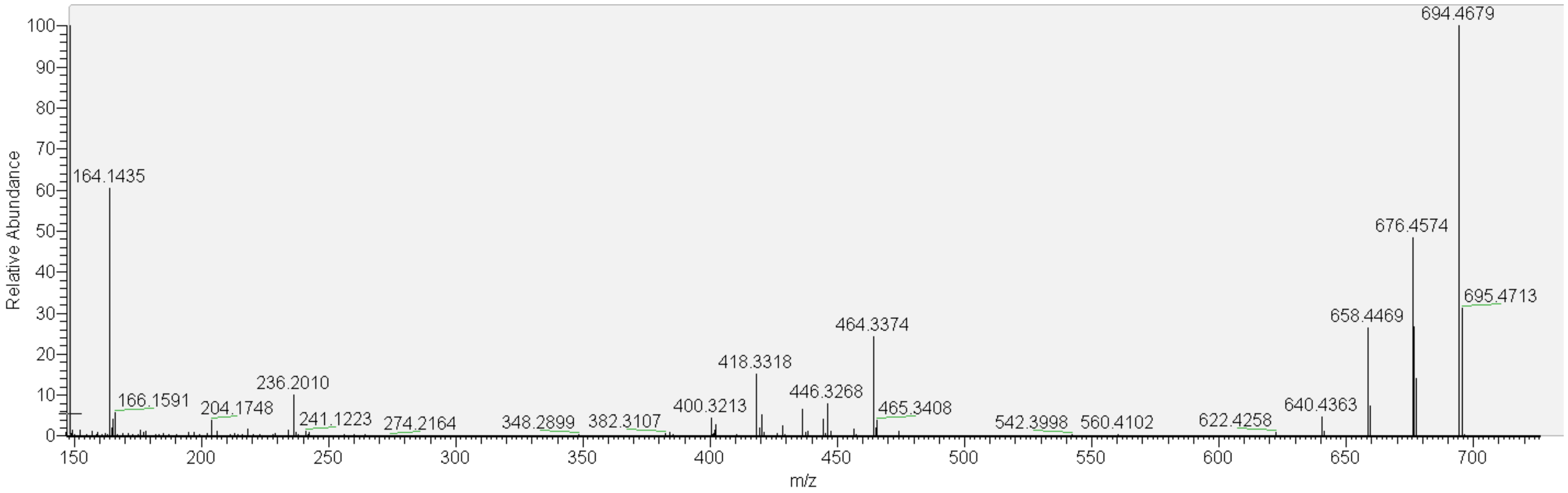

Figure S5. HRMS/MS spectrum of compound **5** (694>164) from MX-S-B11.

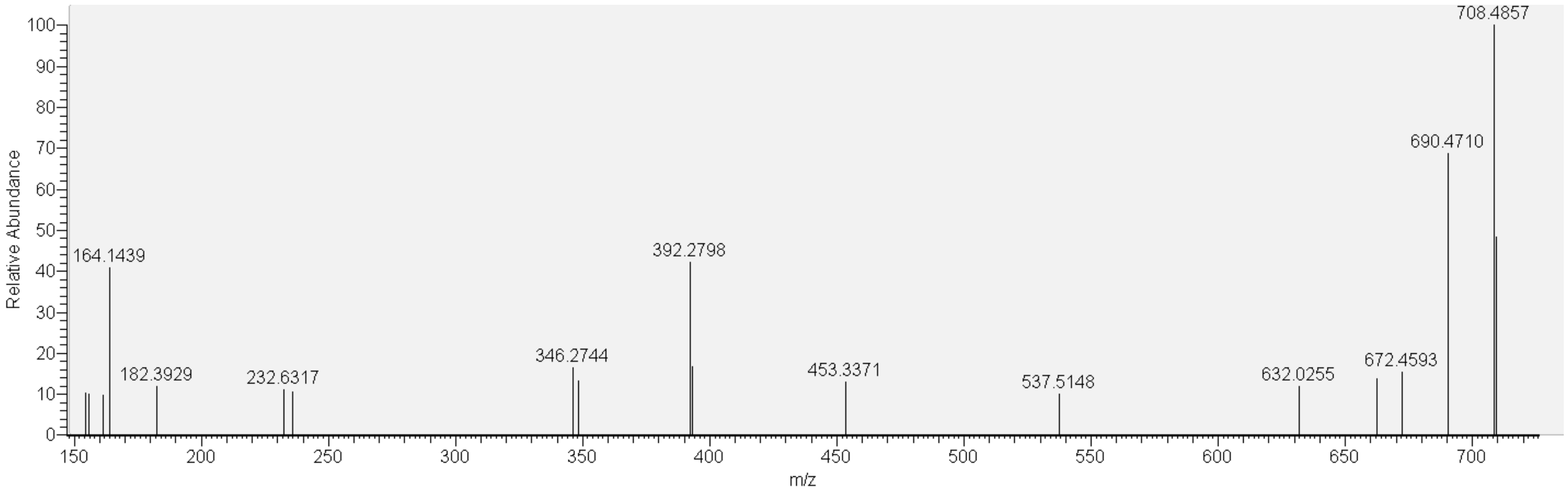

Figure S6. HRMS/MS spectrum of compound **6** (708>164) from MX-S-B11. The 708.4857 peak in the HRMS/MS spectrum displayed an uneven shape. The m/z 690 and 672 peaks are clearly two water losses that do not fit the 708.4857 value with a reasonable ppm error. They, however, do perfectly fit the 708.4836 ion observed in the full scan. Therefore, we argue that the m/z 708.4857 reading in the MSMS scan is created by two unresolved peaks and used the m/z value from the full scan for the calculation of the elemental formula of the pseudo-molecular ion.

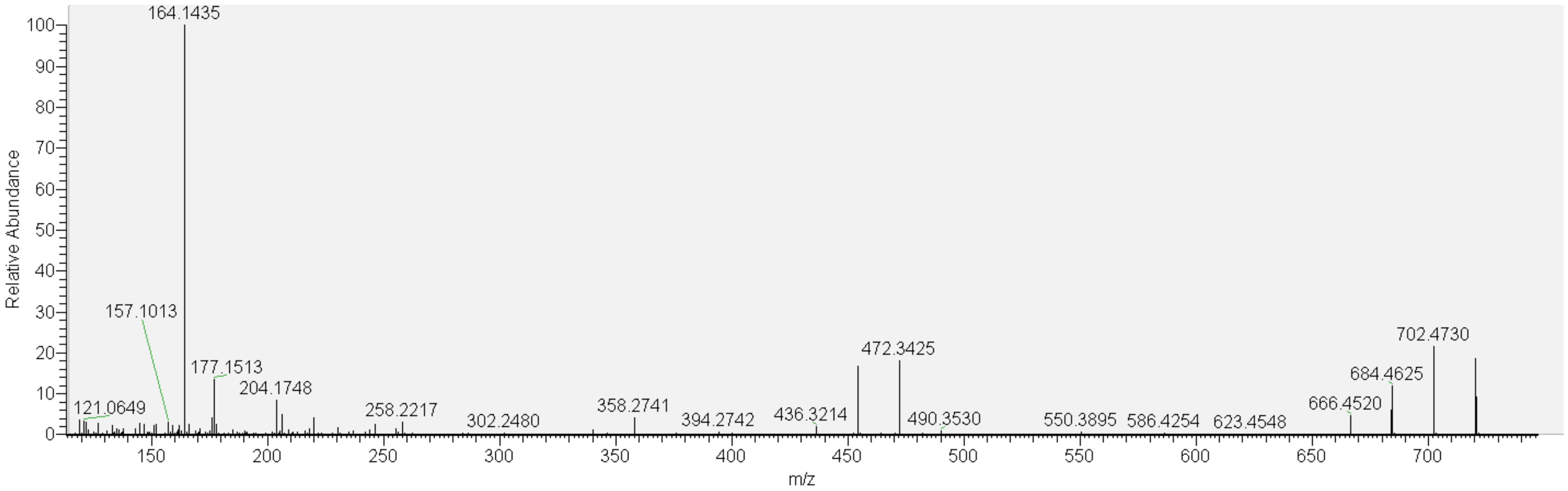

Figure S7. HRMS/MS spectrum of compound **7** (720>164) from MX-S-B11.

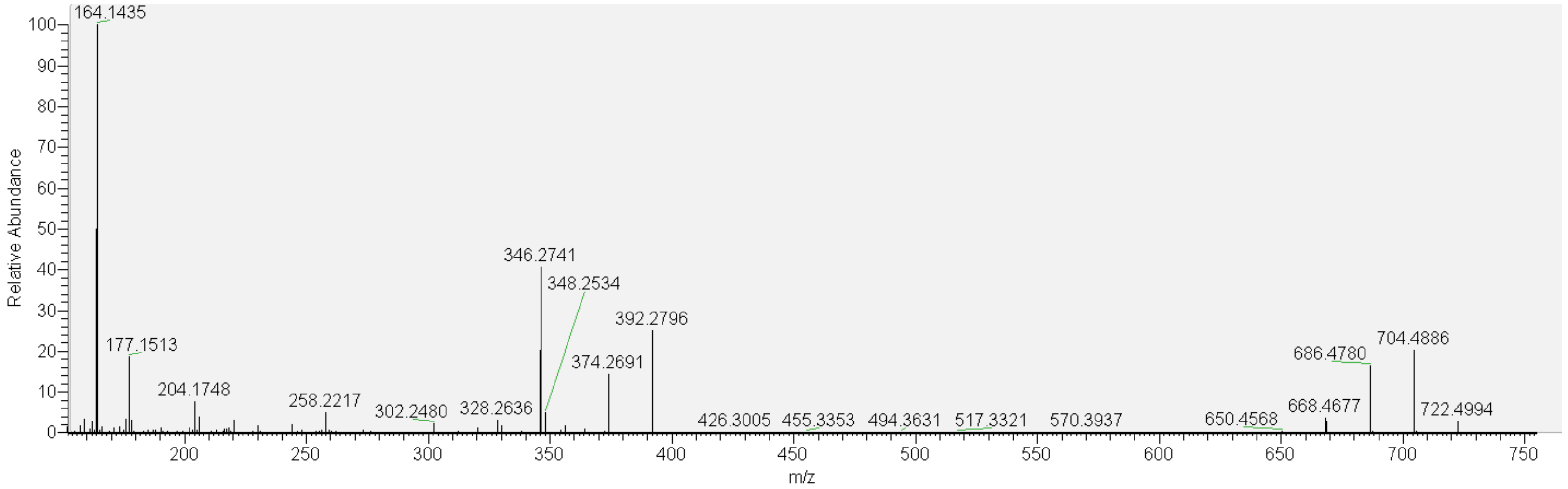

Figure S8. HRMS/MS spectrum compound **8** (678>164) from MX-S-B11.

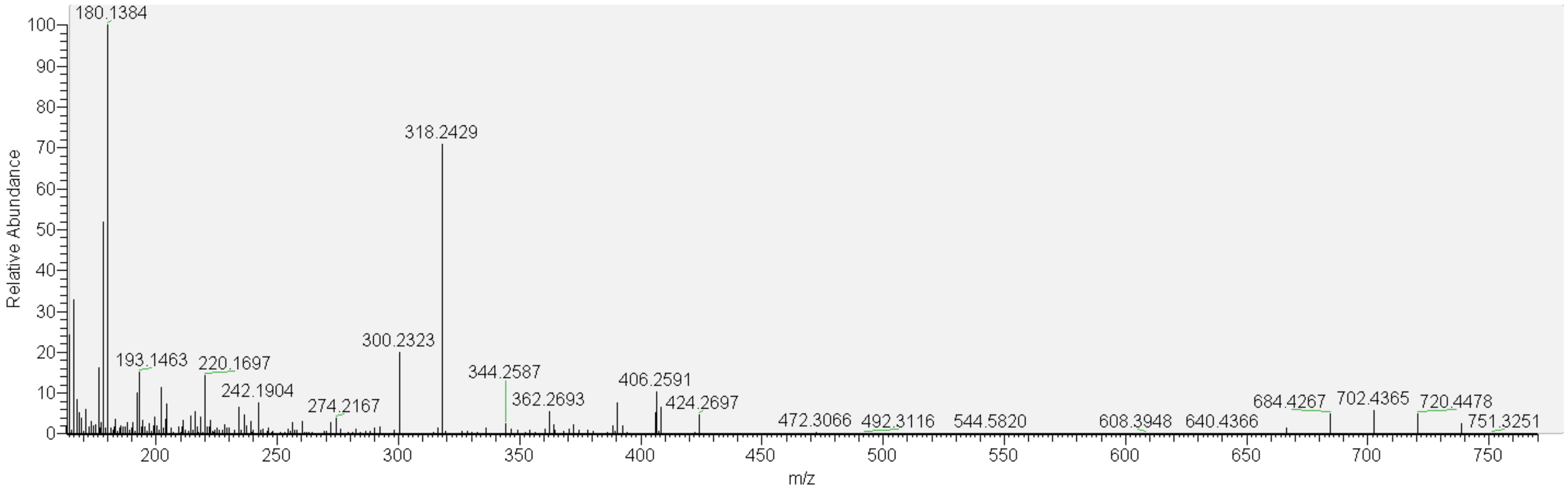

Figure S9. HRMS/MS spectrum compound **9** (738>180) from MX-S-B11.

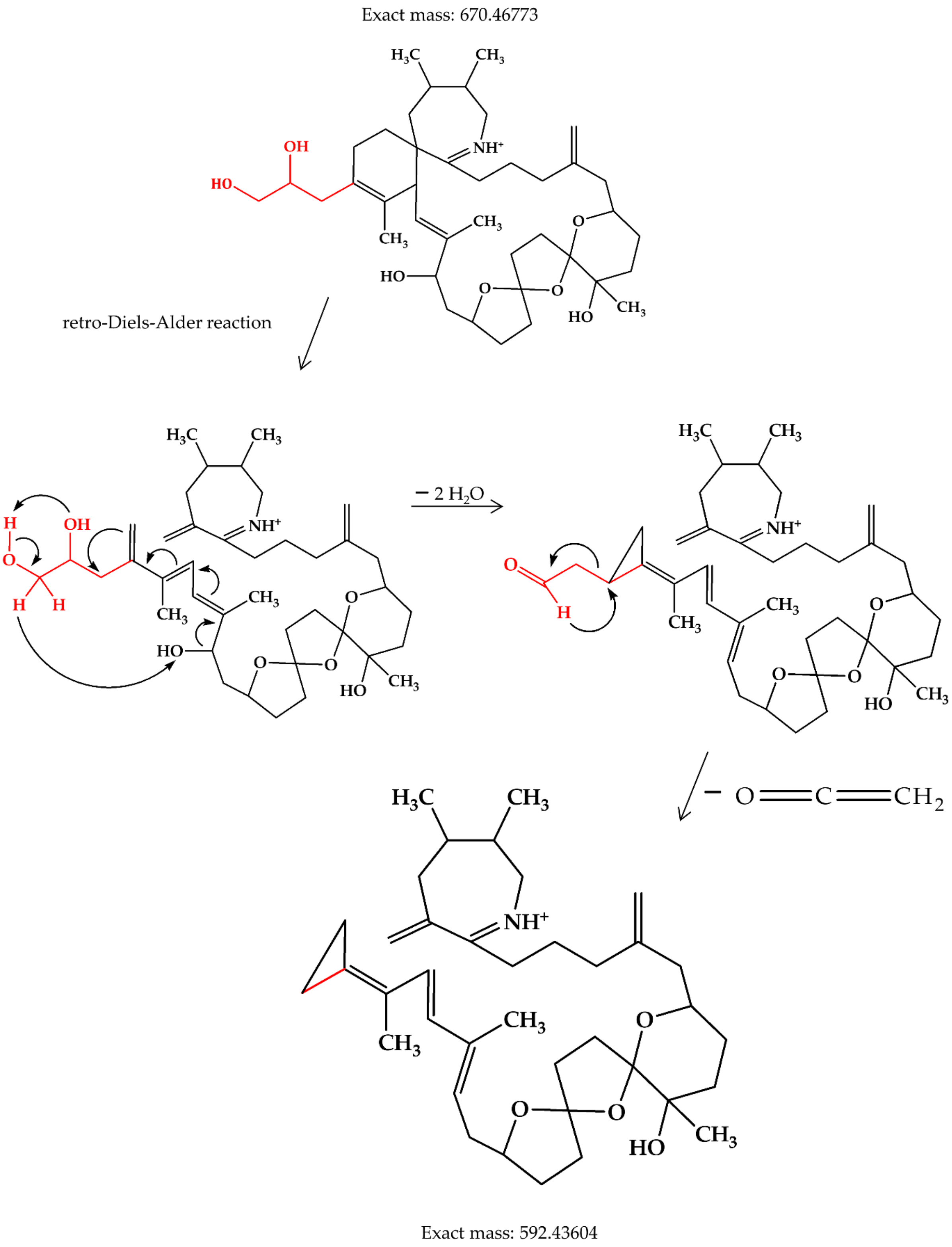

Figure S10. Proposed fragmentation pattern of *m*/*z* 592 from compound **1** (670>164).

Table S1. Extended fragment list from known spirolides.

Spirolide	Mass transition (*m/z*)	Fragments (*m/z*)				Reference
		Group 1	Group 2	Group 3	Group 4	
A	692 > 150	692/674/624	444/390	190	150	[7]
B	694 > 150	694/676/658/640	462/444/426		150	[3-4]
C	706 > 164	706/688/638	458/404	204	164	[7]
13-desMethyl C	692 > 164	692/674/656/638	462/444/426	230	164	[7]
13,19-didesMethyl C	678 > 164	678/660/642/624	448/430/412/394		164	[9,15]
20-Hydroxy-13,19-didesMethyl C	694 > 164	694/676/658/640	446/428/410/392	292/274/248/230	164	[11]
27-Hydroxy-13-desMethyl C	708 > 180	708/690/672/654/636	478/460/442/424		180	[10]
27-Hydroxy-13,19-didesMethyl C	694 > 180	694/676/658/640	464/446/428/410		180	[10]
27-Oxo-13,19-didesMethyl C	692 > 178	692/674/624	444		178	[10]
D	708 > 164	708/690/672/654	458/440	230/206/204/177	164	[3]
13-desMethyl D	694 > 164	694/676/658/640	444/426	230/204/177	164	[8]
20-Hydroxy-13,19-didesMethyl D	696 > 164	696/678/660/642	446/428/410/392	292/274/248/230	164	[11]
G	692 > 164	692/674/656/638	378/360/332	258	164	[12]
20-Methyl G	706 > 164	706/688/670/652	392/374/346	258	164	[12]
H	650 > 164	650/632/614	402/384	206	164	[13]
I	652 > 164	652/634/616	402/384	206	164	[13]

Table S2. Extended HRMS/MS fragment list of compounds **1** from AON 24.

**Compound 1 (670>164)**					
m/z	Intensity	Relative	Theoretical Mass	Δ (ppm)	Elemental Composition
670.4678	631	9.48	670.4677	0.04	$C_{40}H_{64}NO_7^+$
652.4574	4794	72.00	652.4572	0.23	$C_{40}H_{62}NO_6^+$
634.4470	1451	21.80	634.4466	0.36	$C_{40}H_{60}NO_5^+$
616.4365	890	13.36	616.436	0.49	$C_{40}H_{58}NO_4^+$
592.4363	373	5.60	592.436	0.30	$C_{38}H_{58}NO_4^+$
574.4258	344	5.16	574.4255	0.31	$C_{38}H_{56}NO_3^+$
462.3219	175	2.62	462.3214	0.47	$C_{27}H_{44}NO_{5+}$
444.3111	1087	16.33	444.3108	0.29	$C_{27}H_{42}NO4^+$
426.3006	416	6.25	426.3003	0.30	$C_{27}H_{40}NO_3^+$
230.1904	477	7.17	230.1903	0.07	$C_{16}H_{24}N^+$
177.1513	804	12.07	177.1512	0.06	$C_{12}H_{19}N^+$
164.1435	6659	100.00	164.1434	0.13	$C_{11}H_{18}N^+$

Table S3. Extended HRMS/MS fragment list of compounds **2** and **3** from NX-56-10.

**Compound 2 (666>164)**					
m/z	Intensity	Relative	Theoretical Mass	Δ (ppm)	Elemental Composition
666.4365	4596	12.69	666.4364	0.09	$C_{40}H_{60}NO_{7+}$
648.4259	17328	47.83	648.4259	0.04	$C_{40}H_{58}NO_6^+$
630.4154	20498	56.58	630.4153	0.10	$C_{40}H_{56}NO_5^+$
550.3898	25	/	550.3891	1.36	$C_{35}H_{52}NO_4^+$
532.3787	326	/	532.3785	0.38	$C_{35}H_{50}NO_3^+$
378.2640	966	2.67	378.2639	0.12	$C_{22}H_{36}NO_4^+$
360.2533	7620	21.03	360.2533	-0.02	$C_{22}H_{34}NO_3^+$
332.2584	21586	59.59	332.2584	-0.04	$C_{21}H_{34}NO_2^+$
246.2216	7500	20.70	246.2216	0.02	$C_{17}H_{28}N^+$
177.1513	4933	13.62	177.1512	0.07	$C_{12}H_{19}N^+$
164.1435	36225	100.00	164.1434	0.10	$C_{11}H_{18}N^+$
**Compound 3 (696>164)**					
m/z	Intensity	Relative	Theoretical Mass	Δ (ppm)	Elemental Composition
696.4471	18674	90.33	696.4470	0.09	$C_{41}H_{62}NO_8^+$
678.4366	20673	100.00	678.4364	0.15	$C_{41}H_{60}NO_7^+$
660.426	10407	50.34	660.4259	0.16	$C_{41}H_{58}NO_6^+$
642.4156	3308	16.00	642.4153	0.29	$C_{41}H_{56}NO_5^+$
624.4048	514	2.49	624.4047	0.05	$C_{41}H_{54}NO_4^+$
608.4315	70.3	/	608.43146	0.83	$C_{38}H_{58}NO_5^+$
590.4208	491	2.37	590.4204	0.45	$C_{38}H_{56}NO_4^+$
572.4100	664	3.21	572.4098	0.21	$C_{38}H_{54}NO_3^+$
554.3998	127	0.62	554.3993	0.52	$C_{38}H_{52}NO_2^+$
500.1120	40	0.19	500.1129	-0.89	$C_{31}H_{18}NO_6^+$
448.3060	1961	9.49	448.3057	0.25	$C_{26}H_{42}NO_5^+$
432.3114	43	0.21	432.3108	0.60	$C_{26}H_{42}NO_4$+
430.2954	9046	43.76	430.2952	0.22	$C_{26}H_{40}NO_4^+$
412.2848	1342	6.49	412.2846	0.17	$C_{26}H_{38}NO_3^+$
394.2742	69	0.34	394.2741	0.18	$C_{26}H_{36}NO_2^+$
248.2009	63	0.31	248.2009	0.05	$C_{16}H_{26}NO^+$
246.2217	1776	8.59	246.2216	0.08	$C_{17}H_{28}N^+$
177.1513	1612	7.80	177.1512	0.10	$C_{12}H_{19}N^+$
164.1435	15352	74.26	164.1434	0.15	$C_{11}H_{18}N^+$

Table S4. Extended HRMS/MS fragment list of compounds **4-9** from MX-S-B11.

**Compound 4 (678>150)**					
m/z	Intensity	Relative	Theoretical Mass	Δ (ppm)	Elemental Composition
678.4363	107716	100.00	678.4364	-0.15	$C_{41}H_{60}NO_7^+$
660.4260	93076	86.41	660.4259	0.10	$C_{41}H_{58}NO_6^+$
642.4155	39210	36.40	642.4153	0.17	$C_{41}H_{56}NO_5^+$
624.4049	17080	15.86	624.4047	0.17	$C_{41}H_{54}NO_4^+$
508.3426	1867	1.73	508.3421	0.45	$C_{32}H_{46}NO_4^+$
490.3318	576	0.53	490.3316	0.25	$C_{32}H_{44}NO_3^+$
448.3059	4390	4.08	448.3057	0.19	$C_{26}H_{42}NO_5^+$
430.2953	35422	32.88	430.2952	0.16	$C_{26}H_{40}NO_4^+$
412.2848	13180	12.24	412.2846	0.14	$C_{26}H_{38}NO_3^+$
394.2742	1535	1.43	394.2741	0.14	$C_{26}H_{36}NO_2^+$
380.2584	467	0.43	380.2584	-0.01	$C_{25}H_{34}NO_2^+$
244.2061	1573	1.46	244.2060	0.08	$C_{17}H_{26}N^+$
230.1904	1180	1.10	230.1903	0.09	$C_{16}H_{24}N^+$
216.1747	3648	3.39	216.1747	0.07	$C_{15}H_{22}N^+$
206.1904	3209	2.98	206.1903	0.09	$C_{14}H_{24}N^+$
192.1748	4859	4.51	192.1747	0.11	$C_{13}H_{22}N^+$
190.1591	6194	5.75	190.1590	0.06	$C_{13}H_{20}N^+$
150.1278	77982	72.40	150.1277	0.10	$C_{10}H_{16}N^+$
**Compound 5 (694>164)**					
m/z	Intensity	Relative	Theoretical Mass	Δ (ppm)	Elemental Composition
694.4679	439976	100.00	694.4677	0.24	$C_{42}H_{64}NO_7^+$
676.4574	215774	49.04	676.4572	0.34	$C_{42}H_{62}NO_6^+$
658.4469	113275	25.75	658.4466	0.45	$C_{42}H_{60}NO_5^+$
640.4363	20366	4.63	640.436	0.48	$C_{42}H_{58}NO_4^+$
622.4258	3963	0.90	622.4255	0.60	$C_{42}H_{56}NO_3^+$
614.4571	1110	0.25	614.4568	0.52	$C_{41}H_{60}NO_3^+$
560.4102	1784	0.41	560.4098	0.71	$C_{37}H_{54}NO_3^+$
474.3581	5138	1.17	474.3578	0.65	$C_{29}H_{48}NO_4^+$
464.3374	107168	24.36	464.337	0.76	$C_{27}H_{46}NO_5^+$
456.3476	7674	1.74	456.3472	0.82	$C_{29}H_{46}NO_3^+$
446.3268	33845	7.69	446.3265	0.73	$C_{27}H_{44}NO_4^+$
444.3476	18160	4.13	444.3472	0.77	$C_{28}H_{46}NO_3^+$
436.3425	28540	6.49	436.3421	0.76	$C_{26}H_{46}NO_4^+$
428.3163	10930	2.48	428.3159	0.85	$C_{27}H_{42}NO_3^+$
420.3111	22489	5.11	420.3108	0.55	$C_{25}H_{42}NO_4^+$
418.3318	64135	14.58	418.3316	0.59	$C_{26}H_{44}NO_3^+$
410.3057	1978	0.45	410.3054	0.83	$C_{27}H_{40}NO_2^+$

402.3006	12085	2.75	402.3003	0.74	$C_{25}H_{40}NO_3^+$
400.3213	19423	4.41	400.321	0.63	$C_{26}H_{42}NO_2^+$
382.3107	3077	0.70	382.3104	0.75	$C_{26}H_{40}NO^+$
236.2010	44657	10.15	236.2009	0.35	$C_{15}H_{26}NO^+$
204.1748	16971	3.86	204.1747	0.78	$C_{14}H_{22}N^+$
177.1513	4197	0.95	177.1512	0.76	$C_{12}H_{19}N^+$
164.1435	269143	61.17	164.1434	0.90	$C_{11}H_{18}N^+$
**Compound 6 (708>164)**					
m/z	Intensity	Relative	Theoretical Mass	Δ (ppm)	Elemental Composition
708.4857	119695	100.00	708.4834	1.06	$C_{43}H_{66}NO_7^+$
690.4710	82644	69.05	690.4728	-2.62	$C_{43}H_{64}NO_6^+$
672.4593	18347	15.33	672.4623	-2.96	$C_{43}H_{63}NO_5^+$
392.2798	49950	41.73	392.2795	0.56	$C_{23}H_{38}NO_4^+$
346.2744	18766	15.68	346.2741	0.86	$C_{22}H_{36}NO_2^+$
164.1439	47095	39.35	164.1434	3.30	$C_{11}H_{18}N^+$
**Compound 7 (720>164)**					
m/z	Intensity	Relative	Theoretical Mass	Δ (ppm)	Elemental Composition
720.4836	838216	18.34	720.4834	0.36	$C_{44}H_{66}NO_7^+$
702.4730	1006502	22.02	702.4728	0.30	$C_{44}H_{64}NO_6^+$
684.4625	524711	11.48	684.4623	0.31	$C_{44}H_{62}NO_5^+$
666.4520	219421	4.80	666.4517	0.42	$C_{44}H_{60}NO_4^+$
586.4254	16660	0.36	586.4255	-0.20	$C_{39}H_{56}NO_3^+$
490.3530	43167	0.94	490.3527	0.61	$C_{29}H_{48}NO_5^+$
472.3425	809589	17.71	472.3421	0.70	$C_{29}H_{46}NO_4^+$
454.3318	778851	17.04	454.3316	0.61	$C_{29}H_{44}NO_3^+$
436.3214	91264	2.00	436.3210	0.86	$C_{29}H_{42}NO_2^+$
376.2849	14601	0.32	376.2846	0.77	$C_{23}H_{38}NO_3^+$
358.2741	181103	3.96	358.2741	0.15	$C_{23}H_{36}NO_2^+$
346.2743	14120	0.31	346.2741	0.59	$C_{22}H_{36}NO_2^+$
340.2637	47557	1.04	340.2635	0.71	$C_{23}H_{34}NO^+$
302.2480	17381	0.38	302.2478	0.58	$C_{20}H_{32}NO^+$
246.2217	115447	2.53	246.2216	0.22	$C_{17}H_{28}N^+$
230.1905	76776	1.68	230.1903	0.58	$C_{16}H_{24}N^+$
177.1513	623947	13.65	177.1512	0.59	$C_{12}H_{19}N^+$
164.1435	4570111	100.00	164.1434	0.72	$C_{11}H_{18}N^+$
**Compound 8 (722>164)**					
m/z	Intensity	Relative	Theoretical Mass	Δ (ppm)	Elemental Composition
722.4994	3498	2.98	722.4990	0.50	$C_{44}H_{68}NO_7^+$
704.4886	24066	20.48	704.4885	0.26	$C_{44}H_{66}NO_6^+$
686.4780	20248	17.23	686.4779	0.18	$C_{44}H_{64}NO_5^+$

686.4420	1734	1.48	686.4415	0.73	$C_{43}H_{60}NO_6^+$
668.4677	4385	3.73	668.4673	0.56	$C_{44}H_{62}NO_4^+$
668.4315	1014	0.86	668.4310	0.76	$C_{43}H_{58}NO_5^+$
650.4568	371	0.32	650.4568	0.12	$C_{44}H_{60}NO_3^+$
392.2796	31295	26.63	392.2795	0.17	$C_{23}H_{38}NO_4^+$
374.2691	16917	14.39	374.2690	0.28	$C_{23}H_{36}NO_3^+$
372.2897	660	0.56	372.2897	-0.09	$C_{24}H_{38}NO_2^+$
364.2846	1170	1.00	364.2846	-0.12	$C_{22}H_{38}NO_3^+$
356.2585	2172	1.85	356.2584	0.22	$C_{23}H_{34}NO_2^+$
354.2794	701	0.60	354.2791	0.87	$C_{24}H_{36}NO^+$
348.2534	5914	5.03	348.2533	0.37	$C_{21}H_{34}NO_3^+$
346.2741	47722	40.60	346.2741	0.24	$C_{22}H_{36}NO_2^+$
302.2480	2632	2.24	302.2478	0.48	$C_{20}H_{32}NO^+$
258.2217	6122	5.21	258.2216	0.32	$C_{18}H_{28}N^+$
230.1904	2108	1.79	230.1903	0.45	$C_{16}H_{24}N^+$
177.1513	22196	18.89	177.1512	0.51	$C_{12}H_{19}N^+$
164.1435	117532	100.00	164.1434	0.72	$C_{11}H_{18}N^+$
**Compound 9 (738>180)**					
m/z	Intensity	Relative	Theoretical Mass	Δ (ppm)	Elemental Composition
738.4579	5015	2.43	738.4576	0.44	$C_{43}H_{64}NO_9^+$
720.4478	10345	5.01	720.4470	1.14	$C_{43}H_{62}NO_8^+$
702.4365	12580	6.09	702.4364	0.13	$C_{43}H_{60}NO_7^+$
684.4267	10002	4.85	684.4259	1.22	$C_{43}H_{58}NO_6^+$
666.4155	2887	1.40	666.4153	0.34	$C_{43}H_{56}NO_5^+$
424.2697	10171	4.93	424.2694	0.68	$C_{23}H_{38}NO_6^+$
406.2591	21182	10.26	406.2588	0.80	$C_{23}H_{36}NO_5^+$
362.2693	11554	5.60	362.2690	0.79	$C_{22}H_{36}NO_3^+$
336.2537	3168	1.53	336.2533	1.11	$C_{20}H_{34}NO_3^+$
318.2429	147628	71.51	318.2428	0.42	$C_{20}H_{32}NO_2^+$
258.1852	2081	1.01	258.1852	0.00	$C_{17}H_{24}NO_+$
242.1904	16377	7.93	242.1903	0.43	$C_{17}H_{24}N^+$
236.2010	9959	4.82	236.2009	0.67	$C_{15}H_{26}NO^+$
234.1854	13794	6.68	234.1852	0.72	$C_{15}H_{24}NO^+$
230.1905	2085	1.01	230.1903	0.91	$C_{16}H_{24}N^+$
230.1540	2970	1.44	230.1539	0.42	$C_{15}H_{20}NO^+$
180.1384	206431	100.00	180.1383	0.59	$C_{11}H_{18}NO_+$
